# Characterization of the CrbS/R Two-Component System in *Pseudomonas fluorescens* Reveals a New Set of Genes under Its Control and a DNA Motif Required for CrbR-Mediated Transcriptional Activation

**DOI:** 10.3389/fmicb.2017.02287

**Published:** 2017-11-20

**Authors:** Edgardo Sepulveda, Andrei N. Lupas

**Affiliations:** Department of Protein Evolution, Max Planck Institute for Developmental Biology, Tübingen, Germany

**Keywords:** two-component signal transduction systems, *Pseudomonas fluorescens*, crbS, CBRA, acetyl-coenzyme A synthetase, acetate metabolism, acetyl-CoA, STAC

## Abstract

The CrbS/R system is a two-component signal transduction system that regulates acetate utilization in *Vibrio cholerae, P. aeruginosa*, and *P. entomophila*. CrbS is a hybrid histidine kinase that belongs to a recently identified family, in which the signaling domain is fused to an SLC5 solute symporter domain through aSTAC domain. Upon activation by CrbS, CrbR activates transcription of the *acs* gene, which encodes an acetyl-CoA synthase (ACS), and the *actP* gene, which encodes an acetate/solute symporter. In this work, we characterized the CrbS/R system in *Pseudomonas fluorescens* SBW25. Through the quantitative proteome analysis of different mutants, we were able to identify a new set of genes under its control, which play an important role during growth on acetate. These results led us to the identification of a conserved DNA motif in the putative promoter region of acetate-utilization genes in the Gammaproteobacteria that is essential for the CrbR-mediated transcriptional activation of genes under acetate-utilizing conditions. Finally, we took advantage of the existence of a second SLC5-containing two-component signal transduction system in *P. fluorescens*, CbrA/B, to demonstrate that the activation of the response regulator by the histidine kinase is not dependent on substrate transport through the SLC5 domain.

## Introduction

The ability to sense and respond to subtle environmental changes is a crucial trait of all organisms. This has led to the evolution of sophisticated systems that recognize environmental stimuli and subsequently trigger specific physiological responses. In bacteria, the two-component signal transduction (TCST) systems regulate chemotaxis, sporulation, nutrient acquisition, and utilization, among other important cellular processes (Hoch, 2000; Stock et al., 2000; Gao and Stock, 2009; Gotoh et al., 2010; Zschiedrich et al., 2016). As stated by their name, Two-component systems are comprised of two elements, a histidine kinase as the sensing and signal transmission component (membrane-bound or cytosolic) and a cytosolic response regulator as the output element. In transmembrane TCST systems, signal detection by an extracytoplasmic sensor domain triggers conformational changes, which propagate across the membrane to the histidine kinase. This results in the phosphorylation of a conserved histidine in the dimerization and histidine phosphotransfer domain (DHp), by the catalytic ATP-binding domain (CA). In many kinases, additional cytoplasmic sensor domains, such as PAS and GAF, regulate this process (Taylor and Zhulin, 1999; Martinez et al., 2002; Gao and Stock, 2009). Finally, a conserved aspartate in the receiver domain of the response regulator (REC) receives the phosphate group.

Recently, our group described the STAC domain; a new protein module associated with bacterial signal transduction (Korycinski et al., 2015). Structurally, the domain consists of a four-helical bundle, composed of two α-hairpins connected by a central loop of typically nine residues; it can be found as a stand-alone protein or within the context of multidomain proteins. In the latter case, it is typically coupled N-terminally to a transmembrane domain related to the sodium-solute symporter family 5 (SLC5) and C-terminally to an array of different domains characteristic of signaling systems, like histidine kinases or the diguanylate cyclase GGDEF domain. Although, the members of the SLC5 family have a variable number of transmembrane domains, those associated with STAC always have thirteen. The role of the STAC domain is still unknown. We speculate that it could mediate interactions with other proteins or regulate the flow of substrates through the transport domain. STAC-containing signaling proteins are broadly represented in bacteria but absent from Archaea and Eukaryotes (Korycinski et al., 2015). Two STAC-containing TCST have been experimentally characterized so far.

CbrA is a histidine kinase that functions as a global regulator of metabolism, virulence, and antibiotic resistance in the *Pseudomonadaceae* (Nishijyo et al., 2001; Zhang and Rainey, 2007; Yeung et al., 2011, 2014; Quiroz-Rocha et al., 2017). Its cognate response regulator, CbrB, possesses a σ54-interacting domain, which regulates expression of different metabolic genes. For example, the CbrAB system governs the utilization of histidine and other amino acids in *P. fluorescens* (Zhang and Rainey, 2007, 2008; Zhang et al., 2015) and glucose uptake in *Azotobacter vinelandii* (Quiroz-Rocha et al., 2017). The CbrAB system also participates in carbon catabolite repression control (Görke and Stülke, 2008). For example, when *P. aeruginosa* and *P. putida* grow on less-favored compounds, the activity of the CbrAB system is enhanced. This leads to an increase in the abundance of the non-coding small RNAs crcZ and crcY, that inhibit the activity of the Crc global regulator, resulting in an increased expression of its targets which participate in catabolism, pathogenesis, resistance to antibiotics, and biofilm formation (O'Toole et al., 2000; Linares et al., 2010; Abdou et al., 2011). Metabolomic studies suggest that the CbrAB system senses the carbon/nitrogen ratio as a way to read carbon limitation, but the actual signaling molecules and the molecular mechanisms of action remain unknown (Valentini et al., 2014; Zhang et al., 2015).

CrbS is a hybrid histidine kinase, which regulates acetate utilization in *Vibrio cholerae, P. aeruginosa*, and *P. entomophila* (Hang et al., 2014; Jacob et al., 2017). CrbR, the cognate response regulator, is a LuxR-family transcriptional activator, which induces expression of the *acs* gene, which encodes an acetyl-CoA synthase (ACS). This enzyme catalyzes the ligation of acetate and coenzyme A (CoA) to produce acetyl-CoA, which subsequently can be fed into the TCA cycle to produce energy and electron carriers. It is proposed that in pathogenic bacteria, the resulting switch from secreting acetate to assimilating it leads to the depletion of intestinal acetate in the host (Hang et al., 2014; Jacob et al., 2017). A RNAseq analysis of *V. cholerae* highlighted several genes involved in diverse pathways, including pathogenesis, as being influenced by this system (Hang et al., 2014). Nevertheless, besides the acetyl-CoA synthase and the acetate transporter ActP no direct link with other genes has been established (Zaoui et al., 2012; Jacob et al., 2017).

**Figure 1:**
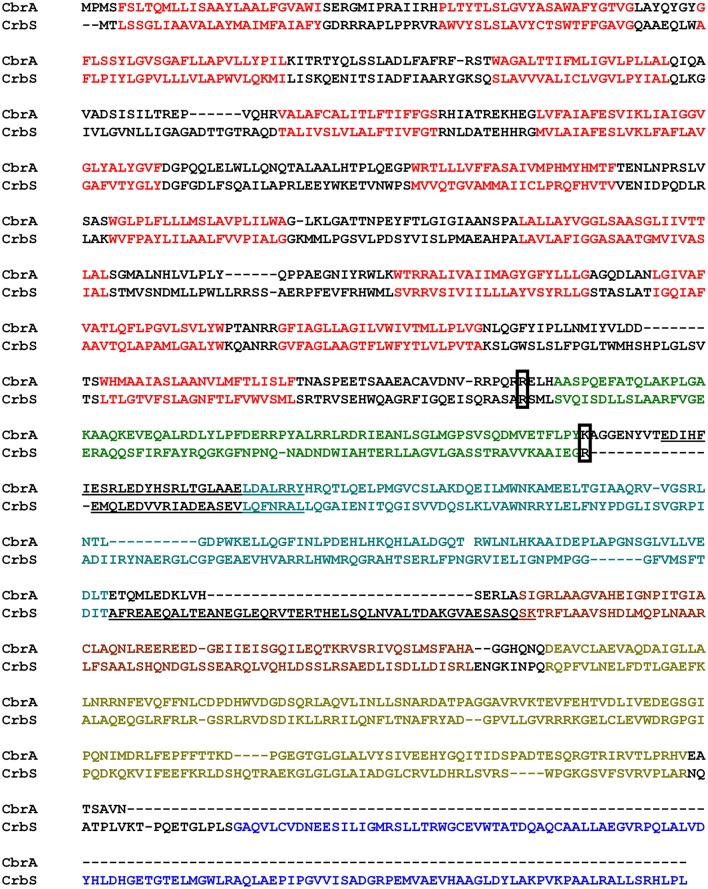
Alignment of CbrA and CrbS from *P. fluorescens*. Relevant features are marked as follows: Red, Transmembrane regions of the SLC5 domain; Green, STAC domain; Light Blue, PAS domain; Brown, DHp domain; Gold, CA domain; Dark Blue, REC Domain. Underlined bases indicate Coiled Coils. Boxed residues indicate the conserved Arginine used as fusion point in the construction of chimeric proteins and deletion points for the STAC domain.

Just as CbrA, CrbS is composed of an SLC5 domain with thirteen transmembrane segments, linked to a histidine kinase by a STAC domain. CrbS differs structurally from CbrA through the REC domain at its C-terminal end of the histidine kinase and through its coiled-coil segments. In particular, the STAC and DHp domains of CrbS are linked by an S-helix, a specific type of dimeric parallel coiled-coil involved in signal transduction (Anantharaman et al., 2006; Stewart and Chen, 2010), which is absent in CbrA (Figure 1).

In this work we use *P. fluorescens* SBW25 as a model to identify new genes in the CrbS/R regulon. Further, using transcriptional fusions, we describe an imperfect palindrome in the promoter region of CrbR-regulated genes that is necessary for transcriptional activation. Finally, we begin the functional characterization of the STAC domain and the STAC-containing signal tranduction systems through the phenotypic analysis of deletion mutants and chimeric proteins.

## Methods

### Growth conditions

*P. fluorescens* and *Escherichia coli* strains were grown in Luria-Bertani (LB) medium at 28 and 37°C, respectively. M9 medium was supplemented with acetate, glucose, or histidine at final concentrations of 2.5–20 mM. When required, antibiotics were added at the following concentrations: ampicillin (Ap), 100 μg ml−1; gentamicin (Gm), 25 μg ml−1; kanamycin (Km), 30 μg ml−1. For selection against pK18mobSacB, LB agar plates were supplemented with 10% sucrose. Genes cloned in pSRKgm were induced by the addition of 100 μM of isopropyl-β-D-thiogalactopyranoside (IPTG).

Pre-cultures for assays were prepared in a volume of 5 ml as described previously (Zhang et al., 2015). Growth curves were performed using a Synergy H4 Hybrid microplate reader with the Gen5 software (BioTek, Winooski, VT) as described previously (Zhang et al., 2015).

### Plasmid and strain construction

Bacterial strains and plasmids used in this study are listed in Table 1. All molecular techniques were conducted following to standard protocols (Sambrook and Russell, 2001). *Escherichia coli* DH5α was used for general cloning, *E. coli* DH5αλpir was used for cloning in pK18mobSacB, and *E. coli* Bl21 DE3 was used for the heterologous expression of proteins. *P. fluorescens* was transformed by electroporation as described elsewhere (Artiguenave et al., 2006). All constructs were verified by sequencing. Primers used in this study are listed in Table 2.

**Table 1:** Strains and Plasmids used in this work.

**Strain**	**Relevant phenotype**	**Reference**
***Pseudomonas fluorescens***		
SBW25	Wild-type strain	Silby et al., 2009
PBR809	*ΔcbrA*	Zhang and Rainey, 2007
ES01	*ΔcrbS*	This work
ES02	*ΔcrbR*	This work
ES03	*Δacs*	This work
ES04	*ΔcrbS* with Tn7*-crbS*, Gm^r^	This work
ES05	*ΔcrbR* pSRKgm-*crbR*, Gm^r^	This work
ES06	*Δacs* pSRKgm*-acs*, Gm^r^	This work
ES07	*Δpflu1813*	This work
ES08	*ΔactP*	This work
ES09	*Δpflu5625*	This work
ES10	*Δ0110*	This work
ES11	*Δpflu1813 Δpflu5625*	This work
ES12	*Δpflu1813 Δ0110*	This work
ES13	*ΔactP Δpflu5625*	This work
ES14	*ΔactP Δ0110*	This work
ES15	*Δpflu5625 Δ0110*	This work
ES16	*ΔactP Δpflu5625 Δ0110*	This work
ES17	*ΔcbrA ΔcrbS*	This work
ES18	*ΔcbrA ΔcrbS* with Tn7-SLC5-CrbS/HK-CbrA, Gm^r^	This work
ES19	*ΔcbrA ΔcrbS* with Tn7-SLC5-CbrA/HK-CrbS, Gm^r^	This work
ES20	*ΔcbrA* with Tn7-*cbrAΔSTAC*, Gm^r^	This work
ES21	*ΔcrbS* with Tn7-*crbSΔSTAC*, Gm^r^	This work
ES22	SBW25 pSRKgm-*crbR*	This work
ES23	SBW25 pSRKgm*-acs*	This work
**Plasmid**	**Relevant phenotype/use**	**Reference**
pSRKgm	Broad-Host-Range Expression Vector, Gm^r^	Khan et al., 2008
pSRKgm-*crbR*	Expression of *crbR* in *P. fluorescens*, Gm^r^	This work
pSRKgm-*acs*	Expression of *acs* in *P. fluorescens*, Gm^r^	This work
pET30b	High-level expression of target proteins. Kmr	Novagen
pET30-crbR	Heterologous expression of CrbR, Km^r^	This work
pK18mobSacB	Allelic exchange vector, Km^r^, Sac^s^	Schäfer
pK18mobSacB-crbS	Deletion of *crbS*, Km^r^, Sac^s^	This work
pK18mobSacB-crbR	Deletion of *crbR*, Km^r^, SacB	This work
pK18mobSacB-acs	Deletion of *acs*, Km^r^, SacB	This work
pK18mobSacB-pflu1813	Deletion of *pflu1813*, Km^r^, SacB	This work
pK18mobSacB-actP	Deletion of *actP*, Km^r^, SacB	This work
pK18mobSacB-pflu5625	Deletion of *pflu5625*, Km^r^, SacB	This work
pK18mobSacB-pflu0110	Deletion of *pflu0110*, Km^r^, SacB	This work
pBB53Gus	Transcriptional fusion vector with *uidA* as reporter gene.	Girard et al., 2000
pBB53GFP	Transcriptional fusion vector with GFP as reporter.	This work
pBB53Gus-Pr*acs*	*UidA* Transcriptional fusion of the *acs* promoter	This work
pBB53Gus-Pr*actP*	*UidA* Transcriptional fusion of the *pflu1318*/*actP* promoter	This work
pBB53Gus-Pr*0110*	*UidA* Transcriptional fusion of the *pflu0110* promoter	This work
pBB53Gus-Pr*5625*	*UidA* Transcriptional fusion of the *pflu5625* promoter	This work
pBB53Gus-Pr*acs*100	*UidA* Transcriptional fusion of the Pr*acs*-100 promoter derivative	This work
pBB53Gus-Pr*acs*-62	*UidA* Transcriptional fusion of the Pr*acs*-62 promoter derivative	This work
pBB53Gus- Pr*acs*-50	*UidA* Transcriptional fusion of the Pr*acs-*50 promoter derivative	This work
pBB53Gus-Pr*acs*+1	*UidA* Transcriptional fusion of the Pr*acs*+1 promoter derivative	This work
pBB53Gus-Pr*acs*-100-18	*UidA* Transcriptional fusion of the Pr*acs*-100-18 promoter derivative	This work
pBB53Gus-Pr*acs*-62+1	*UidA* Transcriptional fusion of the Pr*acs*-62+1 promoter derivative	This work
pBB53Gus-Pr*acs*1M	*UidA* Transcriptional fusion of the Pracs1M promoter derivative	This work
pBB53Gus-Pr*acs4M*	*UidA* Transcriptional fusion of the Pracs4M promoter derivative	This work
pBB53GFP-Pr*acs*	GFP Transcriptional fusion of the *acs* promoter	This work
pBB53GFP-Pr*acs4M*	GFP Transcriptional fusion of the Pr*acs4M* promoter derivative	This work
pUX-BF13	Helper plasmid for transposition of the Tn7element, Ap^r^	This work
pEntr221	Intermediate plasmid for Gateway cloning, Ampr	Invitrogen
*pCR8-cbrA*	Intermediate plasmid for Gateway cloning of cbrA	Zhang et al., 2015
pUC18T-mini-Tn7T-Gm-GW	Chromosome integrative broad-range cloning and expression, Gm^r^	Choi et al., 2005
pUC18T-mini-Tn7T-crbS	Expression of crbS, Gm^r^	This work
pUC18T-mini-Tn7T-Chim1	Integration of chimeric construct SLC5-CrbS/HK-CbrA, Gm^r^	This work
pUC18T-mini-Tn7T-Chim2	Integration of chimeric construct SLC5-CbrA/HK-CrbS, Gm^r^	This work
pUC18T-mini-Tn7T-STACa	Expression of *crbSΔSTAC*, Gm^r^	This work
pUC18T-mini-Tn7T-STACS	Expression of *crbSΔSTAC*, Gm^r^	This work

**Table 2:** Primers used in this work.

**Name**	**Sequence**	**Use**
CrbS-800f	CTGCTCTAGACGACGAGGAATTCGAATTGC	Cloning of the *crbS* region in pK18mobSacB
CrbS-800R	CTGCTCTAGACCTGGCTAAGGAGCTTGAACG	Cloning of the *crbS* region in pK18mobSacB
CrbSdel1	GGTAACTCCGAGCATAGGAACAC	Deletion of *crbS*
CrbSdel1	GTCAGTGCCACATTTTGGTTTGTAC	Deletion of *crbS*
CrbSGWF	GGGGACAAGTTTGTACAAAAAAGCAGGCTTCGACAACGCCCTTTC	Gateway cloning of the *crbS* region
CrbSGWR	GGGGACCACTTTGTACAAGAAAGCTGGGTTCAGCCGGACCGAG	Gateway cloning of the *crbS* region
pflu1195-500f	TACTGTCAAGCTTGCAAGCGCCCATTGTTCATCT	Cloning of the *crbR* region in pK18mobSacB
pflu1195-500r	TACTGTCGAATTCCCTGAGGGTCAGACCAGGATCA	Cloning of the *crbR* region in pK18mobSacB
pflu1195del1	ACGGCGATTTTCCGCAAGCT	Deletion of *crbR*
pflu1195del2	GGCTATCAGGATTTCGTATGTGGCCAT	Deletion of *crbR*
pflu1195NdeIF	TACTGTCCATATGGCCACATACGAAATCCTGATAG	Cloning of *crbR* in pSRKgm and pET30
pflu1195BamHIR	TACTGTCGGATCCCTAGTGCTGCGAAATTGACTCAAGT	Cloning of *crbR* in pSRKgm and pET30
4766-500f	GGATCCGAATTCCATCGTCGAATGGTTCTATAGGCC	Cloning of the *crbS* region in pK18mobSacB
4766L-500r	GGATCCAAGCTTACAGCCTCTGGATTTTATGCAGAGAC	Cloning of the *crbS* region in pK18mobSacB
4766del1	ATTGCTTCGCCGGACGTGATC	Deletion of *crbS*
4766del2	CACCACCGAGTGAATCGCACC	Deletion of *crbS*
4766UNdeI	GAATTCCATATGATGAGTGCGGCTTCCCTGTAC	Cloning of *acs* in pSRKgm
4766LBam	GAATTCGGATCCTTACGCGACGTTCATGGTCTT	Cloning of *acs* in pSRKgm
1813-500f	CTATTGAATTCCGCTTCCTGGACGATCCAA	Cloning of the *pflu1813* region in pK18mobSacB
1813-500r	CTATTGGATCCGGTAGTCCAGGCCGAACAGC	Cloning of the *pflu1813* region in pK18mobSacB
1813del1	TGTTTTTATCCTCGCAGCACAGC	Deletion of *pflu1813*
1813del2	ATGATCCGGCGTCTACTGGC	Deletion of *pflu1813*
actP-750f	CTATTGAATTCAGTCGCGGCCTTCCTGAA	Cloning of the *actP* region in pK18mobSacB
actP-750r	CTATTGGATCCCGGCGATGTGTCGAACATC	Cloning of the *actP* region in pK18mobSacB
actPdel1	TTGCGCAGCCTCCTTGAG	Deletion of actP
actPdel2	AGGTTGCAGCTGGATAAAGAAATGC	Deletion of *actP*
5625-750f	CTATTGAATTCCACCGCAGAATCAGGACGC	Cloning of the *pflu5625* region in pK18mobSacB
5625-750r	CTATTGGATCCCAAGCCCGCTCACTACGAGG	Cloning of the *pflu5625* region in pK18mobSacB
5625del1	CATAACAGTGACCGCAATTTTTTTGTC	Deletion of *pflu5625*
5625del2	AGTTTGTTCGGCTGGCTGC	Deletion of *pflu5625*
0110-700f	CTATTGAATTCCTCGGCGGTTTCGGC	Cloning of the *pflu0110* region in pK18mobSacB
0110-700r	CTATTGGATCCAAAGCCCGCCTCCGGT	Cloning of the *pflu0110* region in pK18mobSacB
0110del1	GGATTCTTATCTCGGGCTACGGA	Deletion of *pflu0110*
0110del2	TGATTGATACAGATCCGAGCTGATG	Deletion of *pflu0110*
Pracs1EcoF	TATCGTGAATTCTATTTACCTTCTTCAGGGCGAAAGG	Cloning of the promoter region of *acs* in pSRKgm
Pracs1kpnR	TATCGTGGTACCCTTTCTTACCTCGGTGACATAGTTGTTGTT	Cloning of the promoter region of *acs* in pSRKgm
Pr5625ecoF	TATCGTGAATTCGGTAAATCAGGCTCCAGCA	Cloning of the promoter region of *pflu5625* in pSRKgm
Pr5625kpnR	TATCGTGGTACCAACAGTGACCGCAATTTTTTTGTCG	Cloning of the promoter region of *pflu5625* in pSRKgm
Pr1813ecoF	TATCGTGAATTCGATTTATGGTGCTGCTGAAACCG	Cloning of the promoter region of *pflu1813* in pSRKgm
Pr1813kpnR	TATCGTGGTACCTGTTTTTATCCTCGCAGCACAGC	Cloning of the promoter region of *pflu1813* in pSRKgm
Pr0110ecoF	TATCGTGAATTCCAAGTGTTCGAGATGGAAGACAT	Cloning of the promoter region of *pflu0110* in pSRKgm
P30110kpnR	TATCGTGGTACCGGATTCTTATCTCGGGCTACGGAA	Cloning of the promoter region of *pflu0110* in pSRKgm
Acs1PrMapF50	TCTGATCGAATTCCTACCATCGTCGAATGGTTCTATAGG	Mapping of the *acs* promoter
Acs1PrMapF100	TCTGATCGAATTCGCTAGAGGTGCAGGAGGGGATA	Mapping of the *acs* promoter
Acs1PrMapR	GAAGGTAAATAGAATTCCTGCAGCC	Mapping of the *acs* promoter
−76	TCTGATCGAATTCTCAGGGCAATTTGTAGGGGC	Mapping of the *acs* promoter
−62	TCTGATCGAATTCTAGGGGCTTGTTACTACCATCGTCG	Mapping of the *acs* promoter
MapfrontF	CCGAGGTAAGAAAGGGTACCCG	Mapping of the *acs* promoter
Map+1R	CAGTATCGGTACCTGGCCCTGTTGTAGCCGG	Mapping of the *acs* promoter
Map+17R	CAGTATCGGTACCCATAGTTGTTGTTGTATGGCCCTGT	Mapping of the *acs* promoter
Map-18R	CAGTATCGGTACCGGCTGGCCTATAGAACCATTCGA	Mapping of the *acs* promoter
MidMut1	GGGCTGGCCTATAGAACCATTGCCAGATGGTAGTAACAAGCCCCTA	Mapping of the *acs* promoter
MidMut2	TAGGGGCTTGTTACTACCATCTGGCAATGGTTCTATAGGCCAGCCC	Mapping of the *acs* promoter
53g-uidAF	CTTTATGCTTGTAAACCGTTTTGTGAAA	Deletion of the *UidA* from pBBR53Gus
53g-uidAR	AGCTGTTTCCTGTGGGGATCC	Deletion of the *UidA* from pBBR53Gus
GFPfwd	ATGAAAGTTAAAGATCTGCGTAAAGGAGAA	Cloning of *gfp* in pBBR53
GFPrev	TTAGTAGTTTTCGTCGTTTGCTGCAGG	Cloning of *gfp* in pBBR53
CbrAGWF	GGGGACAAGTTTGTACAAAAAAGCAGGCTTCTACCTGCAGGAACTGC	Gateway cloning of the *crbrA* region
CbrAGWR	GGGGACCACTTTGTACAAGAAAGCTGGGTTGAAGCATCTCGTCATGG	Gateway cloning of the *crbrA* region
crbsSLC5HybR	GGCAGCAGCATGTCGTTGGA	Chimeric constructs
cbraTCSTHzbF	CGCCAGTGCCCGTGAATTGCATGCCG	Chimeric constructs
cbraSLC5HybR	GGCAGCAGCATGGTCACGATC	Chimeric constructs
crbsTCTSHybF	GTGAAGAACGGGCCCAGCAA	Chimeric constructs
CrbSdelSTAC1	GCGGGCACTGGCGC	Deletion of the STAC domain from CrbS
CrbSdelSTAC2	GAAATGCAGTTGGAGGACGTCG	Deletion of the STAC domain from CrbS
CbrAdelSTAC1	ACGGCGTTGCGGGC	Deletion of the STAC domain from CbrA
CbrAdelSTAC2	GCCGGCGGCGAAAAC	Deletion of the STAC domain from CbrA
ACS1RACE1	ATCGCCCTCCCAGATGATCG	5′ RACE *acs*
ACS1RACE2	GTCCAGGCAGTTGTAGGAAA	5′ RACE *acs*
ACS1RACEseq	ATGTCGACATGGTGATCGTCG	5′ RACE *acs*
ActPRACE1	TTGCGCAGCCTCCTTGAGAATC	5′ RACE *actP*
ActPRACE2	GCCCGACGCACATAGAT	5′ RACE *actP*
ActPRACEseq	AGGTAATCGACGAACCGGGG	5′ RACE *actP*
0110RACE1	CGTTGATCGCTTTGCGCAAGGTA	5′ RACE *pflu0110*
0110RACE2	TGCTTATCCAGGTCATTGC	5′ RACE *pflu0110*
5625RACE1	GTGGTGATTTCGAAGGTGGTTTCA	5′ RACE *pflu5625*
5625RACE2	CCTGCTTGACGATGTAAAAGT	5′ RACE *pflu5625*

*Relevant restriction sites are underlined*.

Plasmids pSRKgm-*acs*, pSRKgm-*crbR*, and pET30-crbR were built by cloning the ORF of each gene into the NdeI/BamHI sites of plasmids pSRKgm and pET30.

Derivatives of pK18mobSacB used for allelic exchange were obtained by cloning in the plasmid a PCR product containing the gene to be deleted, together with 500 bp to 1 kb of upstream and downstream sequence. Then the targeted gene was amplified out by total PCR of the plasmid with phosphorylated divergent primers. The resulting PCR product was ligated, and the religated plasmid was recovered by transformation.

To construct plasmid pUC18T-mini-Tn7T-Gm-GW-*crbS*, the *crbS* coding region with its putative promoter region was PCR-amplified and cloned into the pEntr221 vector from Invitrogen using the Gateway BP Clonase II enzyme mix (Invitrogen). The *crbS* region was then transferred to vector pUC18T-mini-Tn7T-Gm-GW, using the Gateway LR Clonase II enzyme mix (Invitrogen). To construct plasmids pUC18T-mini-Tn7T-Gm-GW-*crbS* ΔSTAC and pUC18T-mini-Tn7T-Gm-GW-*cbrA* ΔSTAC, the STAC domain was amplified from plasmids pEntr221-crbS and pCR8-cbrA respectively, using phosphorylated divergent primers. The resulting PCR product was ligated, and the religated plasmid was recovered by transformation. The *crbS* ΔSTAC and *cbrA* ΔSTAC regions were then transferred to vector pUC18T-mini-Tn7T-Gm-GW, using the Gateway LR Clonase II enzyme mix (Invitrogen).

Chimeric constructs Pr*crbS*-SLC5(CrbS)/HK(CbrA) and Pr*cbrA*-SLC5(CbrA)/HK(CrbS) were obtained by overlapping PCR of a synthetic DNA fragment and two PCR products as illustrated in Figure S1 and in Supplementary File 1. The resulting product was cloned in pUC18T-mini-Tn7T-Gm-GW with pEntr221 as an intermediary using gateway technology as described earlier in this section.

Plasmid pBBGFP was constructed by amplifying out the uidA gene of pBB53Gus with divergent primers. The resulting PCR was ligated with a phosphorylated PCR product of the GFP gene from pET3a[GFP(LVA); Ferris et al., 2012]. Transcriptional fusions were constructed by cloning a PCR product of each putative promoter region in the EcoRI/KpnI sites of plasmid pBB53Gus or pBB53GFP. Mutagenesis of the *acs* promoter transcriptional fusions was achieved by the quick-change strategy (Agilent) using plasmid pBBR53Pr*acs*GUS or pBBR53*acs*GFP as a template. Transcriptional fusions used to map the *acs* promoter were built by amplifying plasmid pBBR53Pr*acs*GUS with divergent primers carrying an EcoRI site or a KpnI site. The resulting PCR product was digested with the corresponding restriction enzyme, ligated, and transformed for the recovery of the reconstituted plasmid.

Strains ES01, ES02, ES03, ES07, ES08, ES09, and ES10 were constructed by allelic exchange selected by sucrose sensitivity using the corresponding derivative of plasmid pK18mobSacB (Schäfer et al., 1994). The same strategy was used for the double and triple mutants in the following combinations. Strains SES11 and ES12 were derived from strain ES07, strains ES13, and ES14 were obtained from strain ES08, strain ES15 was constructed from strain ES09, strain ES16 was generated from strain ES13, and strain ES17 from strain PBR809.

Strains ES04 and ES21 were built through Tn7 mediated integration by transforming strain ES01 with pUX-BF13 and the corresponding derivative of vector pUC18T-mini-Tn7T-Gm-GW as describe elsewhere (Choi et al., 2005; Choi and Schweizer, 2006). The same strategy was used for strains ES18 and ES19, which were derived from strain ES17, and for strains ES20 and ES21 derived from strain PBR809 and strain ES01, respectively.

### Proteomic analysis

Five-milliliter cultures of strains sbw25, ES01, and ES03 were inoculated from pre-cultures to an A_620_ of 0.5 in M9 medium supplemented with acetate and incubated at 30°C. After 8 h cultures were pelleted, frozen with liquid nitrogen and delivered to the Proteome Center of the University of Tübingen for processing and analysis. After tryptic digestion and methylation, samples were examined by liquid chromatography-tandem mass spectrometryLC-MS/MS analysis on a Proxeon Easy-nLC1200 coupled to a QExactive HF (130 min gradient, HCD, Top12). Processing of data was done using MaxQuant software (vs. 1.5.2.8). The spectra were searched against a UniProt- *Pseudomonas fluorescens* SBW25 database (7,063 entries). Down-stream analysis was done using Perseus software (vs. 1.5.0.15) to perform significanceB calculation of normalized protein group ratios (*P*-value threshold 0.01). Dataset is available as Supplementary File 2.

### β-glucuronidase assays

Five-milliliter cultures of the appropriate strains were inoculated from pre-cultures to an A_620_ of 0.5 in M9 medium supplemented with glucose or acetate and incubated at 30°C for 8 h. β-Glucuronidase activity expressed in modified Miller units was quantified as described previously (Jefferson et al., 1986). Photometric measurements were performed with the Synergy H4 Hybrid microplate reader.

### GFP detection assays

Five-milliliter cultures of the appropriate strains were inoculated from pre-cultures to an A_620_ of 0.5 in LB medium, and heterologous expression of CrbR was induced with 500 μM IPTG (isopropyl-β-D-thiogalactopyranoside). After 4 h of incubation at 37°C, 200 μl of each culture were transferred to a 96-well plate, and GFP fluorescence (485/530 nm), and cell density A_620_ were measured using the Synergy H4 Hybrid microplate reader.

### 5′race

RNA was isolated using the Rneasy Mini kit (Qiagen) from a 5 ml culture of P. fluorescens SBW25 (O.D.600 0.5) incubated under acetate-utilizing conditions for 8 h. cDNA synthesis was performed with the RevertAid First-Strand cDNA Synthesis Kit (ThermoScientific) using primers ACS1RACE1, ActPRACE1, 0110RACE1, and 5625RACE1 for *acs, pflu1813/actP, pflu0110*, and *pflu5625*, respectively. A poly-ATP tail was added to the 5′ end of cDNAs using Terminal Transferase (ThermoScientific) as instructed by the manufacturer and the tailed cDNA sample was subjected to PCR amplification using kit's oligo(dT)_18_ primer and primers ACS1RACE2, ActPRACE2, 0110RACE2, and 5625RACE2. Only PCR reactions for *acs* and *pflu1813/actP* generated products of the expected size. Finally, the PCR products were sequenced using primers ACS1RACEseq and ActPRACEseq.

### Bioinformatics and statistical analysis

The Microbial Genomic Context Viewer (Overmars et al., 2013) was used to search for homologs of the CrbS/R regulon in Gammaproteobacteria and to retrieve their putative promoter region. The search was performed in all the genomes available in the platform, but when several strains of the same species were available, only one was used. MEME (Bailey et al., 2009) and FIMO (Grant et al., 2011) searches were run with the default options searching for a motif between 6 and 30 nucleotides long. Sequence analyses were performed in the MPI bioinformatics toolkit (Alva et al., 2016; https://toolkit.tuebingen.mpg.de) using Quick2D, HHpred (Hildebrand et al., 2009), PCOILS (Lupas et al., 1991), and MARCOIL (Delorenzi and Speed, 2002). Transmembrane segments were predicted with FMHMM (Sonnhammer et al., 1998) and Phobius (Käll et al., 2004). For transcriptional fusions, comparisons between samples were performed by ANOVA and Tukey's multiple comparison test using SPSS version 23 (IBM).

## Results

### Identification of proteins regulated by CrbS/R in *P. fluorescens* sbw25

**Figure 2:**
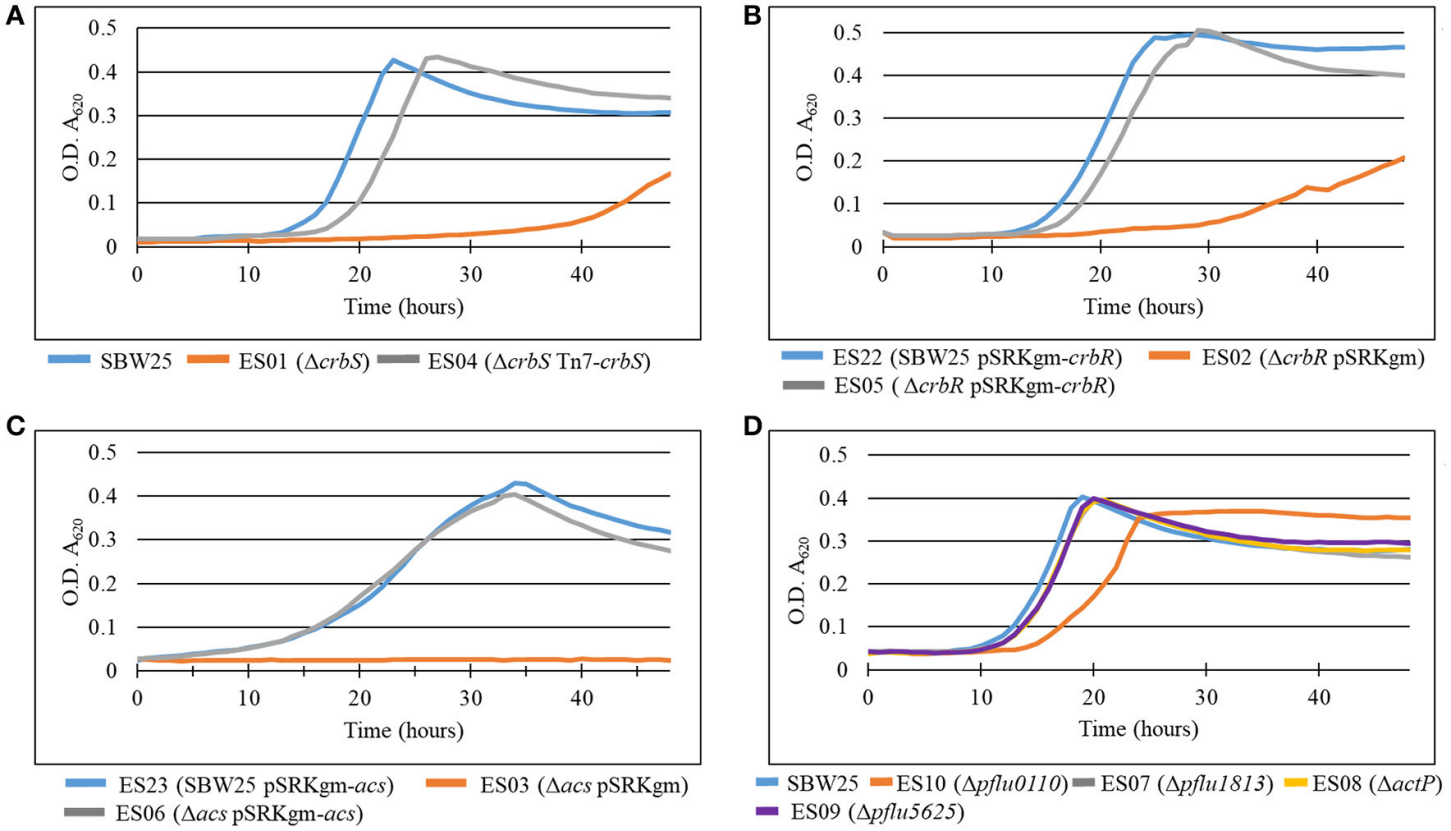
Growth curves of deletion mutants of **(A)** *crbS*, **(B)** *crbR*, **(C)** *acs*, and **(D)** members of the crbS/R regulon in *P. fluorescens* SBW25. Bacteria were grown in M9 minimal medium with acetate as the sole source of carbon. Results are means for six independent cultures. Relevant phenotypes are indicated in parenthesis.

**Figure 3:**
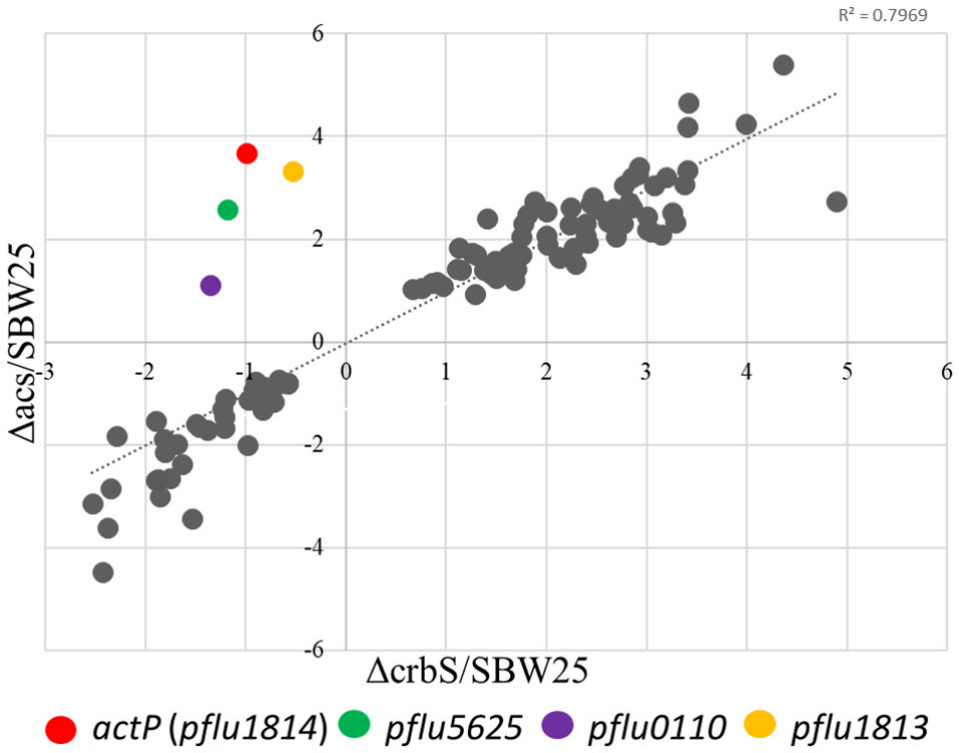
Dispersion graph showing the relative abundances (mutant/WT) of proteins in the Δ*acs* and Δ*crbS P. fluorescens* strains compared to the wild-type strain (SBW25) when growing on acetate. Colored dots indicate proteins in which direction of change rate differed between the Δ*acs* and Δ*crbS* strains. Only proteins with significant changes in abundances (*P* ≤ 0.01) are represented.

In *P. fluorescens* sbw25, CrbS is encoded by the gene *pflu4471*, CrbR by the gene *pflu1195*, and the acetyl-CoA synthase by the gene *pflu4766*. Although, regulation of *acs* by CrbR has been shown in several species of Gammaproteobacteria, there is little knowledge about other possible genes under the control of the CrbS/R system. First, we verified that the behavior of the CrbS/R system in *P. fluorescens* is as reported in other bacteria. To do so, we constructed strains ES01 (Δ*crbS*), ES02 (Δ*crbR*), and ES03 (Δ*acs*) from *P. fluorescens* SBW25 and tested them for their ability to grow on acetate. As expected, the three deletion mutants were unable to grow on M9 minimal medium supplemented with acetate, unless complemented with the corresponding gene (Figures 2A–C). Next, we performed a quantitative proteome analysis of cultures of strains SBW25, ES01 (Δ*crbS*), and ES03 (Δ*acs*) after incubation for 8 h in M9 minimal media supplemented with acetate. This approach permits the direct comparison of protein abundance between strains, allowing us to distinguish those proteins that changed their abundance as a consequence of acetate utilization from those that were affected by the deletion of Δ*crbS*. We identified four proteins, Pflu0110, Pflu1813, ActP, and Pflu5625 that increased their abundance in the SBW25 and ES03 strains but decreased it in strain ES01. Since both mutants are unable to grow on acetate, differences in protein abundance between the two strains can be directly linked to the deletion of CrbS (Figure 3).

**Figure 4:**
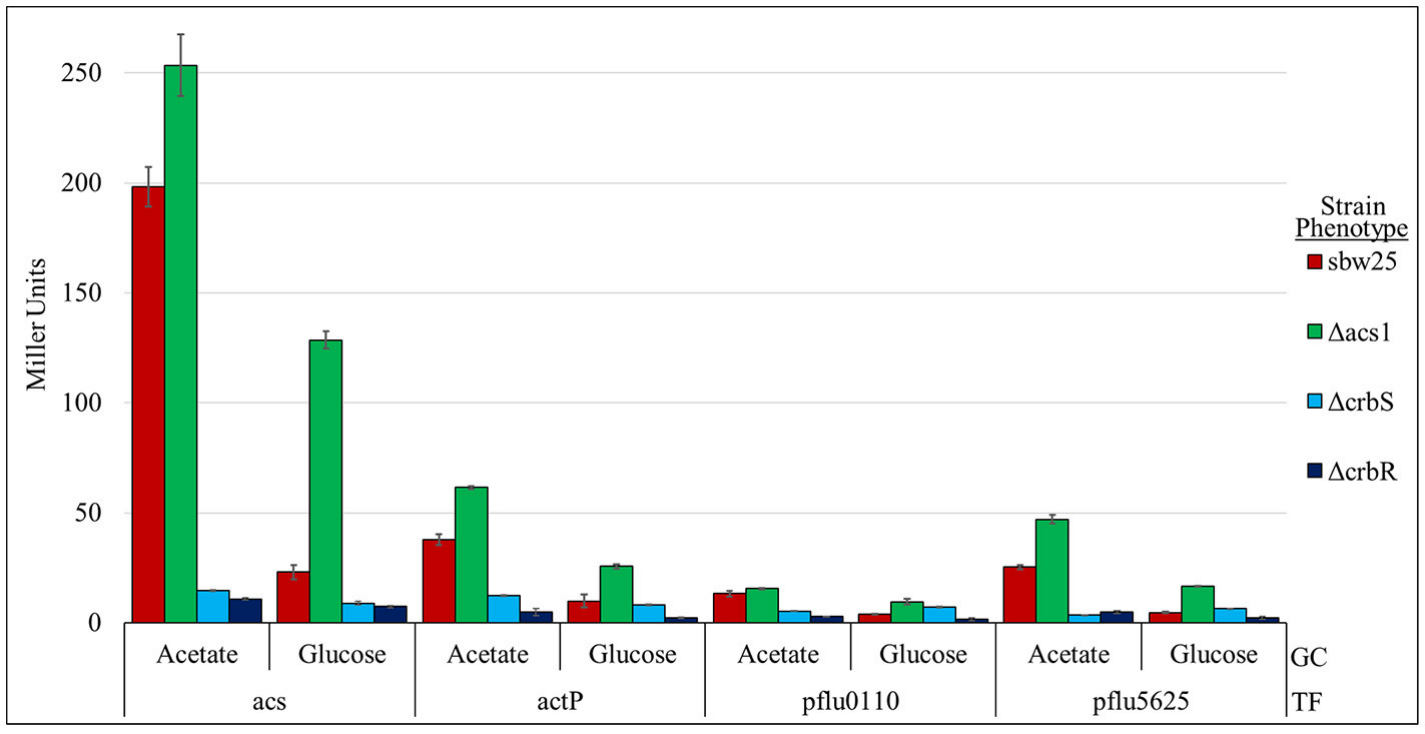
Activity of transcriptional fusions of promoters of genes of the CrbS/R regulon in various genetic backgrounds. Miller units are expressed as means ± Standard deviations (error bars) of results from at least three independent experiments. GC- Growth conditions: M9 minimal medium complemented with 20 mM Acetate or Glucose. TF- Transcriptional fusions of the putative promoter region of the indicated gene. The ANOVA Tukey's *post-hoc* test results for these data can be found in Figure S2.

*actP* has been shown previously to be transcriptionally activated by CrbR (Jacob et al., 2017) and *pflu1813* is found in the same operon as *actP* (*pflu1814*) throughout Gammaproteobacteria. This supports the notion that CrbR also regulates the other genes identified through our approach. To confirm this hypothesis we built β-glucuronidase transcriptional fusions of the putative promoter region of each of the genes that code for the identified proteins, plus *acs*, and tested their expression in different genetic backgrounds. Our results show that all five transcriptional fusions are activated in a wt strain when incubated in M9 medium supplemented with acetate, but not with glucose (Figure 4 and Figure S2). This activation did not happen in the ES01 (Δ*crbS*) nor the ES02 (Δ*crbR*) strains (Figure 4 and Figure S2), demonstrating that the CrbS/R system controls these genes. Interestingly, in strain ES03 (Δ*acs*), all transcriptional fusions showed high levels of transcription when incubated with glucose. This observation is consistent with the results from the proteome analysis. It could be explained by the continuous triggering of the CrbS/R system, present in this strain, because of the accumulation of acetate from the growth medium and the cell metabolism, due to lack of acetyl-CoA synthase activity (Figure 4). Whether acetate or an unknown related molecule is the signal responsible for triggering the CrbS/R system is still pending to be experimentally determined.

### Pflu0110 is necessary for optimal growth in acetate

So far, we have demonstrated that Pflu0110, Pflu1813, ActP, and Pflu5625 are part of the CrbS/R regulon. Pflu0110 is predicted to be an Acyl-CoA Hydrolase, an enzyme that could hydrolyze acetyl-CoA into CoA and acetate. Pflu1813 is annotated as a putative membrane protein of unknown function that belongs to the DUF485 family. ActP is a cation/acetate symporter, which as mentioned previously, has been shown to be part of the CrbS/R regulon. Seeking to understand the role that these proteins have in acetate utilization we constructed deletion mutants of each of the genes and challenged them to grow on M9 minimal media supplemented with acetate. While strains ES07, ES08, and ES09 (Δ*pflu1813*, Δ*actP*, and Δ*pflu5625*, respectively) showed a negligible effect, the strain ES10 (Δ*pflu0110*) presented a 5-h delay in reaching stationary phase, which also occurred at a lower optical density (Figure 2D). The minimal impact of *pflu1813, actP*, and *plfu5625* deletions on acetate utilization could be explained by a suppression effect between them. To discard this possibility, we constructed different combinations of double and triple mutants and tested their ability to grow on acetate. Only those in which *pflu0110* was deleted showed an impairment of their capacity to utilize acetate on the same scale as strain ES10 (Data not shown).

### Characterization of the Crbs/R activated promoters

In the next step, we studied the sequence properties of crbR-responsive promoters that mediate specific activation under acetate utilizing conditions. We used 5′ RACE to determine the transcription initiation site (TIS) from the *acs* and *actP* promoters. These are located at 34 and 36 base pairs from the first codon of the ORF, respectively. In both regions, immediately after the transcription initiation site, we found a Crc motif (AAnAAnAA) that suggests catabolite repression (Moreno et al., 2015). Additionally, in the first 36 base pairs upstream of the initiation sites we located putative RpoD promoters (Potvin et al., 2008), an observation congruent with an earlier bioinformatic analysis in *P. aeruginosa* (Schulz et al., 2015). Although, we did not succeed to identify the transcription initiation site in the case of genes *pflu0110*, and *pflu5625*, we also found putative Crc motifs around 30 nucleotides upstream of the beginning of the ORFs.

**Figure 5:**
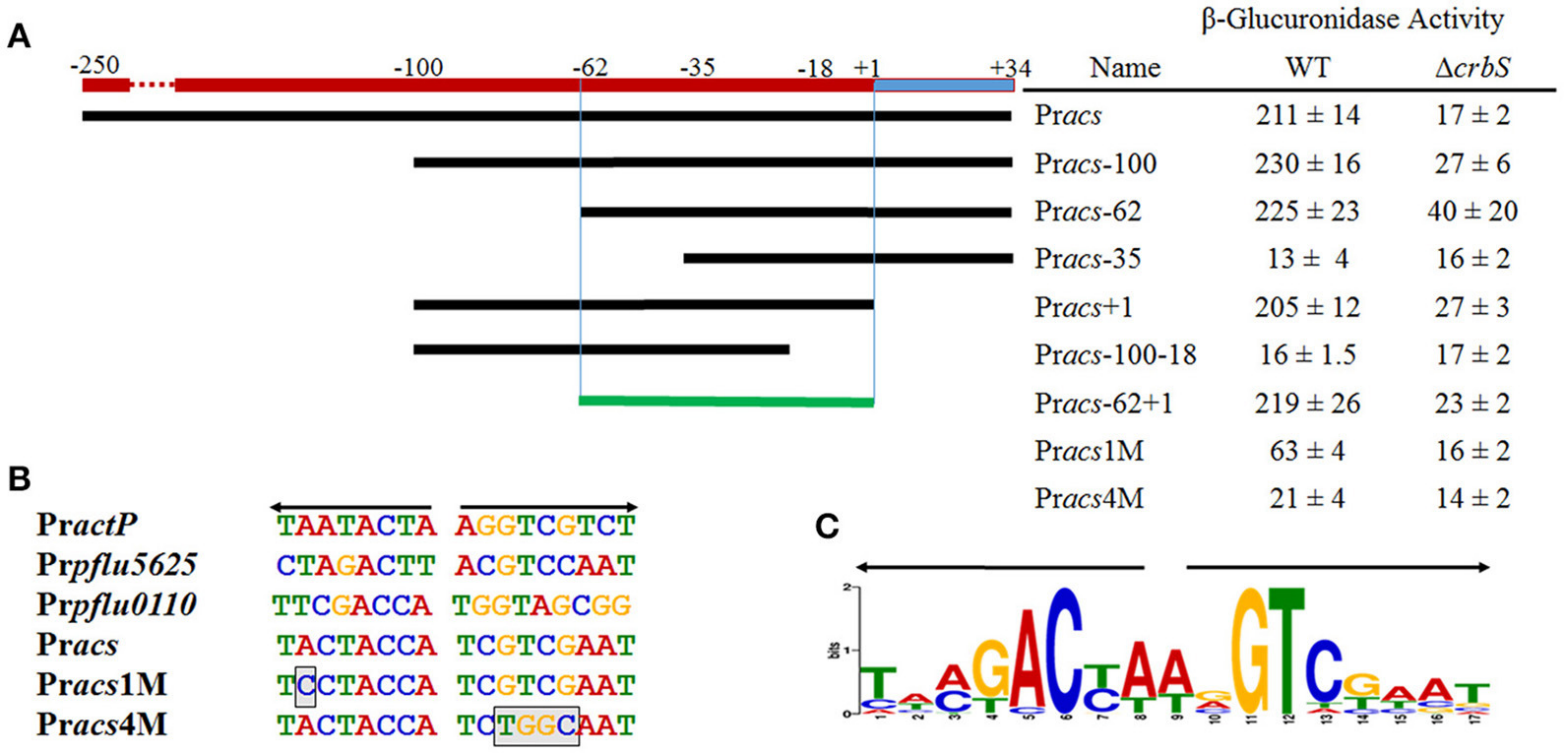
Mapping of the acs promoter and identification of the ACTU motif. **(A)** Activities of transcriptional fusions of derivatives of the *acs* promoter in a wild-type or a Δ*crbS* strain under Acetate-utilizing conditions. Miller units are expressed as means ± Standard deviations of results from at least three independent experiments. (**B**) Sequence of the ACTU motif of the CrbS/R regulon and the *acs* promoter derivatives with one (Pr*acs*1M) and four (Pr*acs*4M) point mutations, respectively, which break the symmetry of the repeat. Boxed bases indicate mutations inserted to disrupt the ACTU motif. **(C)** Sequence Logo of the ACTU motif.

To determine the minimal region necessary for CrbR-dependent regulation, we built transcriptional fusions from several derivatives of the *acs* promoter and measured their activity in M9 medium supplemented with acetate in a wt and a Δ*crbR* background (Figure 5A). Our results outlined a minimal inducible promoter encompassing the region between the −62 and +1 nucleotides of our original construct (Figure 5 fusion Pr*acs*-62+1). Most notably, the dramatic loss of induction of the Pr*acs*-35 derivative that ends just after the predicted RpoD promoter, compared to Pr*acs*-62, indicated that an element essential for CrbS/R-mediated induction could be located in this region (Figure 5 fusion Pr*acs*-62 and Pr*acs*-35).

### An imperfect inverted repeat is required for CrbR-mediated transcriptional activation

**Figure 6:**
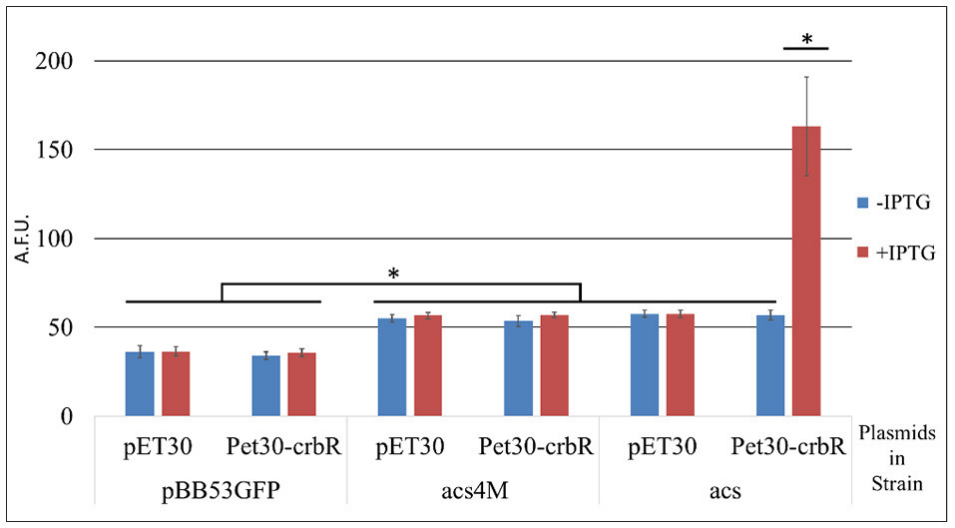
Activity of transcriptional fusions of the *acs* promoter in *E. coli*. BL21. Arbitrary fluorescence units are expressed as means ± standard deviations (error bars) of results from at least three independent experiments. pBB53GFP- promoterless transcriptional fusion vector. *acs*- GFP transcriptional fusion of the *acs* promoter. *acs*4M- GFP transcriptional fusion of the *acs* promoter with four mutations disrupting the ACTU motif. pET30- Empty expression plasmid. pET30-crbR- Plasmid for the induced expression of CrbR. ^*^p < 0.005; ANOVA Tukey's *post-hoc* test.

CrbR is a member of the LuxR family, whose members are known to recognize and bind inverted repeats (Evangelista-Martínez et al., 2006; Antunes et al., 2008). To identify any conserved motifs required for CrbR-mediated promoter induction, we obtained the sequence of the putative promoter region of *acs* homologs from 31 Gammaproteobacteria (Table 3) and ran a motif search using the MEME, a tool for the discovery of novel, ungapped motifs in sequences (Bailey et al., 2009). We were able to identify an imperfect inverted repeat toward the 5′ end of the coding strand, which was present in all the sequences analyzed. Particularly, in the *acs* promoter of *P. fluorescens* SBW25, it is located between the −52 and −36 bases from the transcription initiation site, immediately before the start of the RpoD promoter (Figure 5A). Not only is this observation is congruent with our results from the mapping of the *acs* promoter, but the identified inverted repeat is also located in the putative promoter regions of *actP, pflu5625*, and *pflu0110* (Figure 5B). Moreover, we performed a motif search of the putative promoter regions of a total of 112 identified homologs of *acs, actP, pflu5625*, and *pflu0110* in 31 species of Gammaproteobacteria. We found the imperfect inverted repeat in 91 (81%) of the analyzed sequences (Table 3) and determined a consensus sequence using MEME (Figure 5C). Consistent with the absence of the CrbS/R system in members of the family, the identified motif was not found in the vicinity of the homologs of these genes in enterobacteria. All the evidence we gathered pointed to a role of this inverted repeat in the transcriptional activation of the CrbS/R regulon in the presence of acetate, so we named it the ACTU motif (*acetate utilization*). To test this hypothesis, we constructed transcriptional fusions with two derivatives of the *acs* promoter with one (Pr*acs*1M) and four (Pr*acs*4M) point mutations, respectively, which break the symmetry of the repeat. When introduced into a wt strain and tested under acetate-utilizing, conditions Pr*acs*1M showed reduced induction levels while fusion Pr*acs4M* show no activity (Figures 5A,B). These results confirm the role of the motif in the acetate-dependent induction of the promoter. Nevertheless, we still needed to establish a direct link between the ACTU motif and the induction of the *acs* promoter via CrbR, as we could not discard the possibility that other proteins in *P. fluorescens* were interacting with the motif. To do so, we cloned CrbS in pET30 (pET30-CrbR) and introduced this plasmid in *E. coli* Bl21 DE3 strains carrying (I) a GFP transcriptional fusion to the *acs* promoter (Pr*acs*), (II) the *acs* promoter derivative with the mutagenized palindrome (Pr*acs4M*-GFP), or (III) a promoterless GFP reporter plasmid. When CrbS expression was induced with IPTG the Pr*acs*-GFP transcriptional fusion, but not the Pr*acs*4M-GFP, was activated. Without the addition of IPTG, or in strains carrying the empty pET30, neither fusion was induced (Figure 6). These results demonstrate that expression of the *acs* promoter requires the presence of Crb and that the activation of the *acs* promoter by CrbR is contingent on the presence of the ACTU motif. Activation was independent of the carbon source, as the reporter levels were measured in complex medium. This suggests that CrbR is activated in *E. coli* through an unknown mechanism. Unspecific phosphorylation is a common occurrence in members of the OmpR/LuxR family (Barbieri et al., 2013; Huynh et al., 2015). The fact that the *acs* promoter is transcribed in *E. coli* is not surprising, considering that RpoD from *Pseudomonas* and its promoter consensus sequence are highly similar to those of *E. coli* (Potvin et al., 2008; Schulz et al., 2015).

**Table 3:** Search for the ACTU motif in homologs of genes from the *P. fluorescens* SBW25 CrbS/R regulon in the +, Gene found with the ACTU motif. −, Gene found without the ACTU motif in the promoter region.

**Strain**	***acs***	***actP***	***pflu0110***	***pflu5625***
*Pseudomonas aeruginosa* PAO1	+	+	+	*actP*
*Pseudomonas brassicacearum* NFM421	+	+	+	+
*Pseudomonas entomophila* L48	+	+	+	+
*Pseudomonas fluorescens* SBW25	+	+	+	+
*Pseudomonas fulva* 12X	+	+	NF	+
*Pseudomonas mendocina*	+	+	NF	−
*Pseudomonas monteilii* SB3078	+	+	+	+
*Pseudomonas poae* RE1114	+	+	+	+
*Pseudomonas putida* BIRD-1	+	+	+	+
*Pseudomonas resinovorans*	+	+	+	+
*Pseudomonas stutzeri* A1501	−	+	+	+
*Pseudomonas. syringae* 1448A	+	+	+	+
*Vibrio anguillarum* 775	+	+	NF	+
*Vibrio cholerae* O395	+	+	NF	+
*Vibrio fischeri* MJ11	+	+	NF	+
*Vibrio furnissii* NCTC 11218	+	+	NF	+
*Vibrio vulnificus* CMCP6	+	+	NF	+
*Acinetobacter oleivorans* DR1	+	+	+	*actP*
*Photobacterium profundum* SS9	+	+	NF	+
*Pseudoalteromonas atlantica* T6c	+	+	+	−
*Pseudoalteromonas haloplanktis* TAC125	+	+	+	NF
*Shewanella baltica* BA175	+	+	NF	+
*Shewanella frigidimarina N*CIMB 400	+	+	NF	*actP*
*Shewanella putrefaciens* 200	+	+	NF	+
*Shewanella woodyi ATCC* 5190	+	+	NF	−
*Thalassolituus oleivorans* MIL-1	+	+	NF	*actP*
*Xanthomonas albilineans* GPE PC73	−	+	NF	NF
*Xanthomonas axonopodis pv.citri str*. 306	+	+	NF	NF
*Xanthomonas campestris pv. Campestris*	+	−	+	NF
*Xanthomonas citri* subsp.*citri* Aw12879	+	+	NF	NF
*Xanthomonas fuscans* 4834R	+	+	NF	NF
*Citrobacter rodentium* ICC168	−	−	NF	NF
*Cronobacter sakazakii* BAA-894	−	−	NF	NF
*Dickeya Dadantii* 3937	−	−	NF	NF
*Enterobacter cloacae* EcWSU1	−	−	−	NF
*Escherichia coli* DH1	−	−	−	NF
*Klebsiella pneumoniae*	−	−	NF	NF
*Legionella pneumophila*	−	NF	NF	NF
*Salmonella enterica*	−	−	−	NF

*NF, Gene not found in the genome. actP, Gene transcribed in an operon from the actP promoter*.

Finally, we used the FIMO module of the MEME suite, that finds individual motif occurrences in a sequence database (Grant et al., 2011), to prospect for additional genes regulated by the CrbR/S system in *P. fluorescens* SBW25. Besides the genes identified in this work, the program predicted new occurrences of the palindromic sequence in the upstream region of several genes (Table 4). These predictions should be approached cautiously as the q-value suggests they may not be significant. However, this was the case for *pflu0110*, which we have experimentally proved to be regulated by CrbR. Therefore, experimental verification should be obtained for the most interesting predictions, for example, genes *pflu3808* and *pflu4952*, which code for Isocitrate dehydrogenase and Fumarase, respectively. Both enzymes are part of the TCA cycle, and particularly the Isocitrate dehydrogenase is located at the branching point of the glyoxylate cycle that cells activate when growing on acetate (Wolfe, 2005).

**Table 4:** New genes with a predicted ACTU motif in *P. fluorescens* SBW25.

**Gene**	**Annotation**	***q*-value**	**Matched_ACTU motif sequence**
PFLU4766	Acs Acetyl-coenzyme A synthetase	0.00315	TACTACCATCGTCGAAT
PFLU0447	Methyl accepting chemotaxis protein	0.00315	TACGACTAAAGTCTAAA
PFLU1813	Hypothetical protein co-transcribed with *actP*	0.0196	TAATACTAAGGTCGTCT
PFLU5624	Transcriptional elongation factor greA	0.077	CTAGACTTACGTCCAAT
PFLU5625	Putative Signal-transduction protein	0.077	CTAGACTTACGTCCAAT
PFLU5314	CheV chemotaxis protein	0.239	TACTACCAAAGTCTAAT
rplJ	Ribosomal protein R50	0.345	TAAGACTTACGTCGCCT
PFLU4006	Ribose ABC transporter ATP binding protein	0.373	CTTGACCAAGGTCGATT
PFLU3808	NADP(+) Isocitrate dehydrogenase	0.517	TACGCCTAAAGTCGCAC
PFLU1460	Putative phage regulatory protein	0.517	TAAGACCTTGGTCTATC
dsbA	Thiol:disulfide interchange protein	0.552	TAAAACCTACGTTGAAT
PFLU4471	CrbS	0.6	GTCGACCAAGGTCGTGT
PFLUt81	tRNA-Met	0.633	TAATCCCTTGGTCGTAG
PFLU0110	Acyl-CoA Hydrolase	0.633	CGCTACCATGGTCGAAT
PFLU3395	Hypothetical protein	0.657	TGAGACTATCGTCTAGT
PFLU1212	Polyamine ABC transporter ATP binding protein	0.717	AACGACCTAGGTCCCCT
PFLU4952	Fumarase	0.737	TTTGACCATAGTCGGGT
PFLU4951	Iiron/sulfur-binding oxidoreductase	0.737	TTTGACCATAGTCGGGT
wssB	Cellulose synthase	0.808	CACGCCCTTCGTCGAAG
PFLU4643	Nitrate reductase	0.828	GCCGACCAAGGTCGAA
PFLU3097	Aldehyde dehydrogenase	0.916	TACGACGTCGGTCGAAT
PFLU2727	SAM dependent methyltransferase	0.916	TACGCCCAAGGTCGGCC
PFLU4120	Multi-drug transporter	0.93	CACGCCCAAAGTCTCGG

*Shaded lines indicate genes experimentally identified in this work. The q-value is the minimal false discovery rate at which a motif occurrence is assumed significant. We used a value of q < 0.05 as a threshold for significance*.

### The SLC5 domain of CrbS can replace the SLC5 domain of CbrA

**Figure 7:**
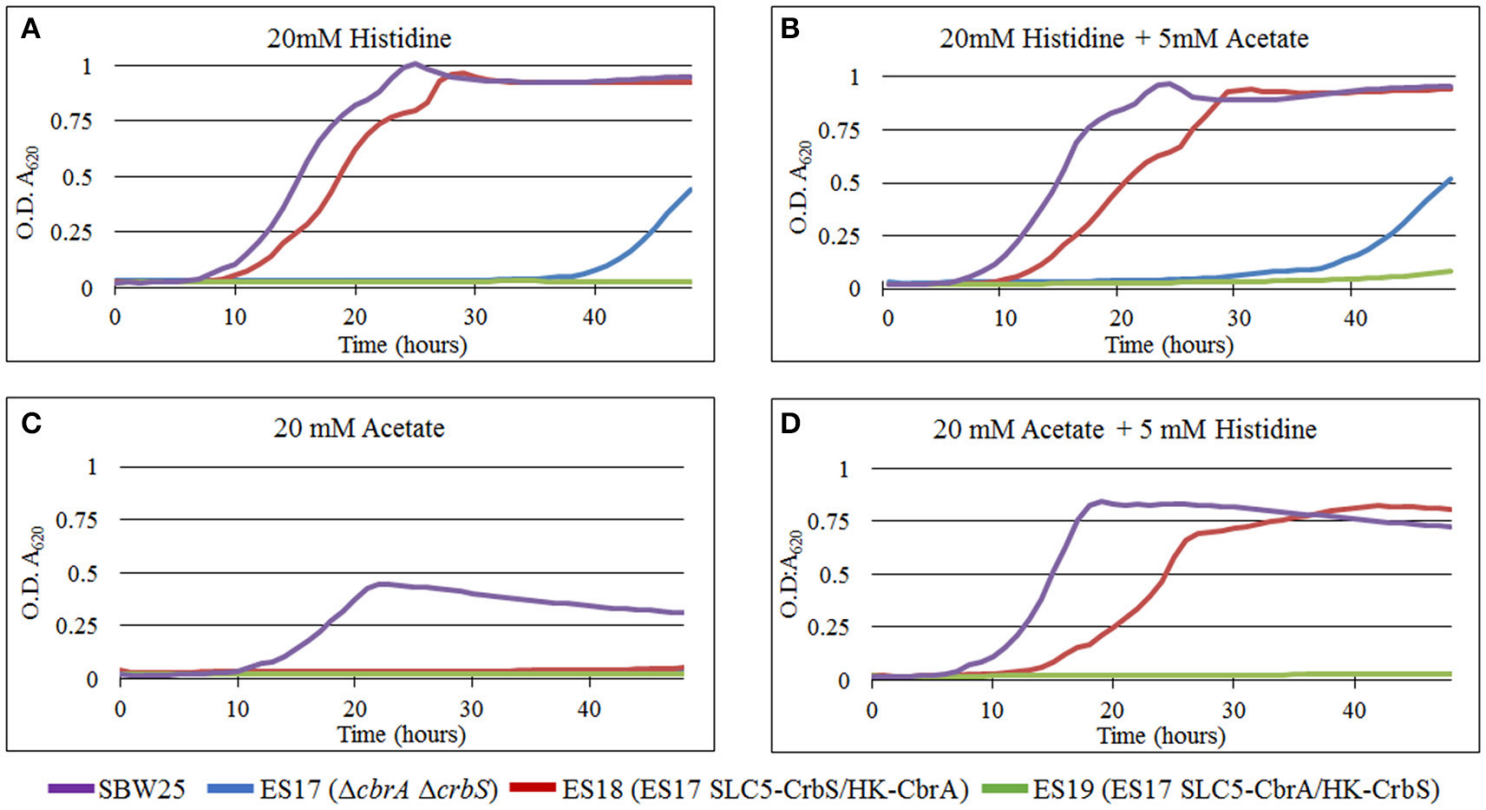
Growth curves of *P. fluorescens* SBW25 strains carrying CrbS/CbrA chimeras. Bacteria were grown in M9 minimal medium complemented with **(A)** 20 mM histidine, **(B)** 20 mM histidine and 5mM acetate, **(C)** 20 mM acetate, and **(D)** 20 mM acetate and 5 mM histidine. Results are means for six independent cultures. Relevant phenotypes are indicated in parenthesis. When histidine is at a 20 mM concentration, it is the only carbon and nitrogen source. Otherwise, ammonium chloride is used as the nitrogen source.

In bacteria, there is mounting evidence for the existence of multi-protein complexes that link transport and signaling. For example, several signal transduction systems use permeases, ABC-transporters, or soluble substrate-binding proteins as co-sensors (Tetsch and Jung, 2009; Västermark and Saier, 2014). In this context, CrbS and CbrA represent a unique type of sensor, in which the signaling and transport domains are connected within the same polypeptide. In the case of CbrA, previous data suggest that the protein not only senses histidine, but is also capable of internalizing it, and that this process is dependent on signaling. Physical coupling between the N-terminal SLC5 domain and the C-terminal Histidine kinase of CbrA is required for function (Zhang et al., 2015). These observations raise the possibility that transport of the substrate through the SLC5 domain elicits a signal, which could trigger or modulate the activity of the histidine kinase. To test this hypothesis, we generated strain ES17, a double Δ*cbrA* Δ*crbS* deletion mutant. When tested, this strain was not able to grow on acetate as a carbon source nor in histidine as a carbon and nitrogen source (Figures 7A–D). Next, using overlapping PCR, we built two chimeric constructs, from CbrA and CrbS, in which the promoter and the SLC5 domain regions of each gene were exchanged. A conserved arginine located immediately before the STAC domain was used as the crossover point (Figure 1 and Figure S1 and Supplementary File 1). These constructs were used independently to complement strain ES17, giving rise to strains ES18 (SLC5-CrbS/HK-CbrA) and ES19 (SLC5-CbrA/HK-CrbS; Figure S1). Both strains were then challenged to grow in M9 media supplemented with histidine or acetate or a combination of both. Strain ES18 was able to thrive in any media containing histidine (Figures 7A,B). The presence of acetate in the medium had no effect, as long as histidine was also added (Figures 7B,D) but the strain was not able to grow solely on it (Figure 7C). These results show that, in CbrA, substrate transport is not needed for the induction of the signal. The previously observed lack of activity of the histidine kinase when not physically coupled to the SLC5 domain can be explained then by increased instability, misfolding, or failure to dimerize. Strain ES19 was not able to grow in any of the conditions tested (Figures 7A–D). Two factors may explain this. (I) The chimera is expressed from the cbrA promoter, which may not be active under acetate-utilizing conditions; (II) CrbS may have additional structural limitations.

### First insights into the role of the STAC domain

Since the STAC domain has been described recently, there are no data yet on its function. To gain an insight into the role of the STAC domain, we constructed strains ES20 (CbrAΔSTAC) and ES21 (CrbSΔSTAC), in which the STAC domain was removed. The limits of the deleted region were carefully designed to avoid disruption of the secondary structure of the protein (Figure 1; boxed residues). Surprisingly, when tested for their ability to utilize the corresponding substrate, there was no obvious difference in growth when compared to the wt strain. Moreover, the activity of the Pr*acs* transcriptional fusion in strain ES21 showed no significant difference under acetate utilizing conditions, when compared to the wt strain (Data not shown).

## Discussion

Despite the characterization of the CrbS/R system and its effect on pathogenesis in different species of Gammaproteobacteria, the study of its role as a regulator of acetate utilization has been limited to its control of acetyl-CoA synthase. In this work, we identified and characterized new genes that are regulated by this system. *Pflu0110* was the only gene whose deletion impaired the ability of *P. fluorescens* to grow on acetate, while deletion of *pflu1318, actP, and pflu5625* had no apparent effect. These genes could, however, play a role under natural conditions. *Pflu0110* is predicted to code for an Acyl-CoA Hydrolase. CoA is the major acyl group carrier in cells, required for the metabolism of carbohydrates, amino acids, fatty acids, and ketone bodies; it is used to modulate the activity of enzymes and transcriptional factors. Under conditions in which acetate is present in high concentration and the main energy source, it is possible to predict a scenario where the intracellular pool of CoA becomes depleted, as it is incorporated into the acetate utilization pathway. As a result, it will be unavailable for other processes that require CoA, hindering cell growth. The activity of the Acyl-CoA hydrolase antagonizes that of the acetyl-CoA synthase, so it is feasible to propose that its role in acetate utilization is to maintain the availability of CoA for other cellular processes. *Pflu1813* encodes a membrane protein of unknown function. However, since it is always found in an operon with *actP*, even in organisms that lack the CrbS/R system, it is probably functionally associated with ActP. ActP was characterized in *E. coli* as an acetate transporter with narrow specificity. As in *P. fluorescens*, an *E. coli* ActP-deficient mutant showed no impairment when growing on acetate (Gimenez et al., 2003). Since cells can transport acetate by passive diffusion, it has been proposed that ActP is required for scavenging acetate in micromolar concentrations (Gimenez et al., 2003). The scale at which we monitor growth on acetate requires concentrations that range between 2.5 and 20 mM, which do not resemble the conditions in nature. As a result, even though we can follow the overall process of acetate utilization, the subtle adjustments and processes in a poor and competitive environment may not be triggered, or are overwhelmed, and go undetected. To circumvent these limitations, we propose the use of experimental setups that better resemble natural conditions, like microcosms (Craig et al., 2004) or the use of infection models, as has been recently done with *P. entomophila* and *Drosophila* (Jacob et al., 2017). The same may explain the lack of phenotype of the *pflu5625* mutant. Pflu5625 is annotated as a signal-transduction protein with cAMP-binding (Berman et al., 2005), CBS (Baykov et al., 2011), and nucleotidyltransferase domains, predicted to regulate protein activity by nucleotidylation in response to changing levels of cAMP, or other unknown molecules (Aravind and Koonin, 1999). A detailed characterization of this protein is required to identify these signals as well as the pathways in which is involved.

The mapping of the promoter regions of *actP* and *acs* allowed us to determine the minimal fragment required for expression and induction of genes controlled by the CrbS/R system. Inside this region, we identified an inverted repeat needed for CrbR-dependent promoter activation, which we named the ACTU motif. *Acs* and *actP* are the only members of the *P. fluorescens* CrbS/R regulon that are conserved among the Gammaproteobacteria while *pflu0110* and *pflu5625* are restricted mostly to the pseudomonales and the vibrionales. Additionally, the ACTU motif was not found in the upstream region of genes of the Enterobacteriaceae, which lack CrbR and CrbS. Altogether, these observations show that, although a conserved set of acetate-utilization genes is present in the Gammaproteobacteria, diversification in accessory genes and regulation has occurred across the lineage.

Chimeras have been proved to be useful tools for the structural and functional characterization of Two-component systems (Utsumi et al., 1989; Skerker et al., 2008; Capra and Laub, 2012; Mondéjar et al., 2012; Ganesh et al., 2013). Our results with the SLC5-CrbS/HK-CbrA chimera show that the histidine kinase domain from CbrA does not depend on a signal triggered by the transport of histidine through the SLC5 domain to activate CbrB. This finding is consistent with the role of these systems in the detection of compounds that are often found in low levels in nature. If signaling depended on transport, under micromolar concentrations of the substrate the activity of the transporters would compete with that of the sensors, risking a premature interruption of induction.We rather propose that the fusion of the SLC5 domain and the histidine kinase in the STAC containing TCST sensors allows for early detection and fast response of subtle concentrations of substrate in a highly competitive environment.

Our tests with the ΔSTAC mutants may have been limited, as discussed earlier, by the scale at which we can measure the ability to utilize carbon sources. The STAC domain is present in all proteins in which an SLC5 domain is linked to the sensor of a Two-component system. This hints for an important role of this domain under conditions that we have not reproduced. We are currently designing new CrbS/CbrA chimeras in order to answer these questions and to continue to pursue the characterization of the processes modulating sensing and signaling in this particular group of proteins.

## Author contributions

ES and AL: Conception and design, acquisition of data, analysis and interpretation of data, writing of the article.

### Conflict of interest statement

The authors declare that the research was conducted in the absence of any commercial or financial relationships that could be construed as a potential conflict of interest.
