# Supplementary material for: Characterization of the CrbS/R Two-Component System in Pseudomonas fluorescens Reveals a New Set of Genes under Its Control and a DNA Motif Required for CrbR-Mediated Transcriptional Activation
Source: Front Microbiol. 2017 Nov 20;8:2287. doi: 10.3389/fmicb.2017.02287 (PMC5715377; doi:10.3389/fmicb.2017.02287)
Supplement: Supplementary file 1 [file Image1.PDF]

Construction of the crbA/cbrA chimeras.

Two PCR products and a synthetic DNA fragment were assembled by overlapping PCR. The external primers have BP Clonase compatible sequence for the introduction of the constructs in the Gateway system. Blue letters correspond to sequence from crbS and green letters to sequence from CbrA. Primer pairs used to amplify PCR products are colored dark or light brown. The arginine used as a fusion point is colored red. The synthetic DNA fragment is in a small case. Promoter regions are in *italic*.

P*crbS*-SLC5(CrbS)/HK(CbrA)

Nucleotide

GGGGACAAGTTTGTACAAAAAAGCAGGCTT*CGACAACGCCCTTTCTGTTGCTCGCCGCCGCCTGCTTCGCTCGAAGCTCCCCGCGCTTCTACCACTG*
GCTGGTGCAACACCCGCGCCTGGGCCCGTGGATCCGTGATTACCTGGACGGCAACGGTATCCCGCTCAAAGGCAAGGTCTACGCCATTGGCTTGATG
TGGCTGAGCATCGGTGTTTCCTGCTACCTGGTGCCCCTGCCGTGGGCGCGTGGTTTCATGCTGACCAGCGCCGTGCTGGTGACACTCTACATCCTGC
GCCAGAAAACCCTGCCACCTCGCTGAGCGCCCCTGCGACATTCGATCCCACACGACCTTGGTCGACACTGACTAACCCCACCCATCATGCGAGACTG
*TCCAACCGGGCCCGGAATGCCTGGGTGTTCCTATGCTCGGAGTTACC*ATGACGCTGTCCAGCGGGCTGATCGCCGCCGTTGCCCTGGCCTATATGGC
CATTATGTTTGCCATCGCCTTCTACGGTGATCGTCGCCGTGCGCCCTTGCCGCCACGTGTACGTGCCTGGGTGTATAGCCTGTCGCTCGCCGTTTAC
TGCACCAGCTGGACCTTCTTCGGCGCCGTAGGCCAGGCTGCCGAACAGCTGTGGGCATTTTTACCGATCTACCTCGGACCGGTTCTATTGCTGGTGT
TGGCACCTTGGGTGCTGCAGAAGATGATTCTGATCAGCAAGCAGGAAAACATCACCTCCATCGCCGACTTTATTGCGGCGCGCTACGGCAAATCCCA
ATCCCTGGCCGTGGTGGTCGCACTGATCTGCCTGGTCGGCGTACTGCCCTATATTGCGCTGCAACTCAAAGGCATCGTGCTGGGAGTCAACCTGCTG
ATCGGCGCGGGTGCCGACACCACCGGCACCCGCGCGCAGGACACCGCACTGATCGTGTCGCTGGTATTGGCGCTGTTCACTATCGTCTTCGGTACCC
GCAACCTCGACGCCACGGAACACCACCGTGGCATGGTGCTGGCGATTGCGTTTGAATCCCTGGTCAAGCTGTTCGCGTTTCTGGCAGTCGGCGCGTT
TGTGACCTACGGCCTGTACGACGGTTTCGGCGACCTGTTCAGCCAGGCCATTCTGGCGCCACGGCTGGAGGAATACTGGAAGGAGACCGTCAACTGG
CCTTCGATGGTGGTGCAGACCGGCGTGGCCATGATGGCGATCATCTGCTTGCCTCGGCAATTTCATGTCACGGTGGTCGAGAACATCGACCCGCAGG
ACCTGCGCCTGGCCAAGTGGGTGTTTCCCGCGTATCTGATTCTTGCCGCGCTGTTTGTAGTACCGATCGCCTTGGGCGGCAAGATGATGCTGCCCGG
CTCGGTGCTTCCGGACTCCTACGTCATCAGCCTGCCGATGGCCGAAGCCCACCCCGCCCTCGCGGTACTGGCATTTATTGGCGGCGCTTCAGCGGCC
ACCGGCATGGTGATCGTGGCGAGCATCgcgctgtcgaccatggtgtccaacgacatgctgctgccgtggttgctgcgtcgctccagcgccgagcggc
cgttcgaagtgttccgccactggatgctctcggtccgccgggtgagcatcgtgatcatcctgttgctggcctacgtcagttaccgcctgctgggttc
gaccgccagcctggcgaccatcggccagatcgcctttgccgccgtgacccaattggcgccggcgatgctcggtgcgctgtactggaaacaggcgaac
cgacgcggtgtattcgcgggcctggcagcgggcaccttcctgtggttctacaccttggtcctaccggtgacggcgaaaagcctgggttggtccctca
gccttttcccaggctgacgtggatgcattcgcacccgctgggcctctccgtcacttcgctgaccctgggcaccgtgttctcactggcgggtaactt
cacgctgtttgtgtgggtgtcgatgctctcgcgcacgcgggtgtccgagcactggcaagccgggcgctttatcggccaggaaatcagccagcgcgcc
agtgcccgtgaattgcatgccgcctcaccccaggagttcgccacgcaactggccaagcctttgggtgccaaagccgcacaaaaggaggtcgaacaag
cgctgcgcgatctctacttgccgttcgacgagcggcgcccttacgcgctgcggcgtctgcgcgaccgtatcgaagccaacctgtccggtttgatggg
acccagtgtgtcccaggacatggtggaaactttcctgccctacaaggccggcggcgaaaactacgtgaccgaagacatccatttcatcgagagccgg
ctcgaggattaccactCACGCCTCACGGGCCTCGCCGCCGAACTCGATGCCCTGCGCCGCTACCACCGCCAGACCCTGCAAGAATTGCCGATGGGCG
TGTGCTCGCTGGCCAAGGATCAAGAGATCCTGATGTGGAACAAGGCCATGGAAGAACTCACCGGTATCGCAGCGCAGCGCGTGGTCGGTTCACGCCT
CAATACCTTGGGCGATCCGTGGAAAGAATTGCTGCAAGGGTTCATCAATCTGCCCGACGAGCACTTGCACAAGCAGCATCTGGCCCTCGACGGCCAA
ACCCGTTGGCTCAACCTGCACAAAGCCGCTATCGACGAGCCCCTGGCGCCCGGTAACAGCGGCCTGGTGTTACTGGTGGAAGACCTGACCGAAACCC
AGATGCTCGAAGACAAGCTGGTGCATTCCGAGCGTCTGGCCAGCATTGGCCGACTGGCGGCGGGCGTGGCCCATGAAATCGGCAACCCGATCACCGG
TATCGCGTGCCTGGCGCAGAACCTGCGCGAAGAGCGCGAGGAAGATGGCGAGATCATCGAGATCAGCGGGCAAATCCTCGAACAGACCAAGCGCGTG
TCACGCATCGTGCAGTCGCTGATGAGCTTTGCCCACGCGGGCGGGCATCAGAACCAAGATGAAGCGGTGTGCCTGGCCGAAGTGGCGCAGGATGCCA
TTGGGCTGCTGGCCTTGAACCGGCGCAATTTCGAAGTACAGTTCTTCAACTTGTGCGACCCCGACCACTGGGTGGACGGCGACTCACAACGCCTGGC
CCAAGTGCTGATCAACCTGCTCTCCAACGCCCGCGATGCAACGCCTGCCGGTGGCGCGGTACGCGTCAAGACCGAGGTTTTCGAACATACGGTCGAT
CTGATCGTCGAAGATGAAGGTAGCGGTATTCCACAGAACATCATGGACCGATTGTTCGAACCCTTCTTCACCACCAAGGACCCAGGTGAAGGTACCG
GTCTGGGCCTTGCACTGGTCTATTCCATCGTTGAAGAGCATTATGGACAAATCACCATCGACAGCCCGGCTGACACCGAAAGCCAACGCGGCACCCG
TATTCGGGTGACCTTGCCGCGTCATGTCGAAGCGACGTCCGCTGTGAACTGAGACCGTCGAGAGAATTGAATCAATGCCGCACATTTTGATCGTCGA
AGACGAAACCATTATCCGCTCTGCCTTGCGTCGCCTGCTTGAACGAAATCAGTACCAGGTCAGCGAAGCCGGCTCGGTGCAGGAAGCCCAGGAGCGT
TTCAGCATTCCCACGTTCGACCTGATTGTCAGTGACCTGCGCCTTCCCGGCGCGCCTGGCACCGAGTTGATCAAGCTTGGCCAGGGCACCCCGGTGC
TGATCATGACCAGCTACGCCAGCCTGCGCTCGGCGGTGGACTCCATGAAGATGGGCGCGGTGGACTACATCGCCAAGCCTTTCGACCATGACGAGAT
GCTTCAACCCAGCTTTCTTGTACAAAGTGGTCCCC

Aminoacid

MTLSSGLIAAVALAYMAIMFAIAFYGDRRRAPLPPRVRAWVYSLSLAVYCTSWTFFGAVGQAAEQLWAFLPIYLGPVLLLVLAPWVLQKMILISKQE
NITSIADFIAARYGKSQSLAVVVALICLVGVLPYIALQLKGIVLGVNLLIGAGADTTGTRAQDTALIVSLVLALFTIVFGTRNLDATEHHRGMVLAI
AFESLVKLFAFLAVGAFVTYGLYDGFGDLFSQAILAPRLEEYWKETVNWPSMVVQTGVAMMAIICLPRQFHVTVVENIDPQDLRLAKWVFPAYLILA

ALFVVPIALGGKMMLPGSVLPDSYVISLPMAEAHPALAVLAFIGGASAATGMVIVASIALSTMVSNDMLLPWLLRRSSAERPFEVFRHWMLSVRRVS
IVIILLLAYVSYRLLGSTASLATIGQIAFAAVTQLAPAMLGALYWKQANRRGVFAGLAAGTFLWFYTLVLPVTAKSLGWSLSLFPGLTWMHSHPLGL
SVTSLTLGTVFSLAGNFTLFVWVSMLSRTRVSEHWQAGRFIGQEISQRASARELHAASPQEFATQLAKPLGAKAAQKEVEQALRDLYLPFDERRPYA
LRRLRDRIEANLSGLMGPSVSQDMVETFLPYKAGGENYVTEDIHFIESRLEDYHSRLTGLAAELDALRRYHRQTLQELPMGVCSLAKDQEILMWNKA
MEELTGIAAQRVVGSRLNTLGDPWKELLQGFINLPDEHLHKQHLALDGQTRWLNLHKAAIDEPLAPGNSGLVLLVEDLTETQMLEDKLVHSERLASI
GRLAAGVAHEIGNPITGIACLAQNLREEREEDGEIIEISGQILEQTKRVSRIVQSLMSFAHAGGHQNQDEAVCLAEVAQDAIGLLALNRRNFEVQFF
NLCDPDHWVDGDSQRLAQVLINLLSNARDATPAGGAVRVKTEVFEHTVDLIVEDEGSGIPQNIMDRLFEPFFTTKDPGEGTGLGLALVYSIVEEHYG
QITIDSPADTESQRGTRIRVTLPRHVEATSAVN

PrcbrA-SLC5(CbrA)/HK(CrbS)

Nucleotide

GGGGACAAGTTTGTACAAAAAAGCAGGCT*TCTACCTGCAGGAACTGCTCGGCCTGCGCCAACCGCGCTACCTGCATGTGCCGCTGATCATCCAGCCG*
GACGGCAACAAGTTGGGCAAATCCTACCGTTCCCCGCCGTTGACACCCGACCAGGCCACGCCTTTGCTATTGAGAGCCCTGCGTGCCCTCGGCCAGC
AACCCGGCGACGAGTTGCTGCACGCCAGCCCACGAGAACTGCTGGACTGGGGCATCCAGCACTGGGATGCCGGCCGGATACCTCGCACACTCACGCT
GGCCGAAGCGCAATTGAGCTGAAGGCGCTTGCAGCTTGCCAGGCATCCGTTACCATCGCCGCAGTTTTTCAATCAGAGGCCAACATGTACATCTATC
GATTGGTCCTGCTGCTGGTCGTCGGGATCTACCTGTTTTCTCCCGCCATCATGGATTGGTGGATCGACGCCACGGGCGCCTGGTATCGCCCCTATCT
*GCTGTGGCTGATCCTGATCGTCGTGACTTTCATCCTGCAGAGCCAAAAAGATGCCGATGAG*CTTTAGCCTCACCCAGATGCTGCTGATCAGTGCCGC
CTACCTGGCCGCGCTGTTCGGGGTGGCCTGGATCAGTGAGCGCGGAATGATTCCGCGGGCGATCATTCGCCATCCGTTGACCTACACCTTGTCCCTG
GGCGTGTACGCCAGTGCCTGGGCGTTCTATGGCACGGTGGGCCTGGCCTATCAATACGGCTATGGCTTTCTTTCCAGTTACCTCGGGGTGTCCGGCG
CGTTCCTGCTGGCACCGGTGTTGCTGTACCCGATCCTGAAAATCACCCGTACCTATCAGCTGTCATCCCTGGCCGACCTGTTTGCCTTCCGCTTTCG
CAGCACCTGGGCCGGCGCGCTGACCACCATTTTCATGCTGATCGGCGTGTTGCCGTTGCTGGCCCTGCAAATCCAGGCGGTGGCTGACTCCATCAGC
ATCCTGACCCGTGAGCCGGTGCAGCACCGCGTTGCCCTGGCTTTCTGCGCGTTGATTACGCTGTTCACGATTTTCTTCGGTTCACGCCATATCGCCA
CTCGCGAGAAACACGAAGGCCTGGTGTTTGCAATTGCCTTCGAGTCCGTGATCAAGCTGATCGCCATCGGCGGTGTCGGCCTTTATGCACTTTACGG
CGTGTTCGACGGCCCGCAGCAGCTTGAGCTTTGGTTGCTGCAAAACCAGACCGCCCTCGCCGCCCTGCACACGCCGCTGCAAGAGGGCCCATGGCGC
ACGCTGCTGCTGGTGTTCTTCGCCTCGGCGATCGTGATGCCACACATGTATCACATGACCTTTACCGAGAACCTCAACCCGCGCTCGCTGGTCAGCG
CCAGCTGGGGTTTGCCGCTGTTCCTGCTGTTGATGAGCCTGGCGGTGCCACTGATCCTGTGGGCAGGCCTGAAACTGGGCGCCACCACTAACCCGGA
ATACTTCACCCTCGGCATCGGCATTGCCGCCAACAGCCCGGCGTTGGCTTTGCTGGCGTACGTCGGTGGTTTGTCAGCCGCCAGCGGGCTGATCATC
GTCACGACCCTGGCGCTCTCGGGCATGGCGCTCAACCATCTGGTGCTACCGCTGTACCAGCCGCCGGCCGAGGGCAACATCTACCGCTGGCTGAAAT
GGACGCGGCGCGCGCTGATCGTCGCGATCATCATGGCCGGCTACGGGTTCTACCTGTTGCTGGGCGCCGGGCAAGACCTGGCCAACCTGGGTATCGT
CGCGTTTGTCGCCACCTTGCAGTTCCTGCCGGGCGTGCTGTCGGTGCTGTACTGGCCGACCGCCAATCGCCGTGGCTTTATCGCCGGCttgctggcg
gggatcctggtgtggatcgtgaccatgctgctgccgctggtcggtaatctgcagggcttctacatcccattgctgaacatgatctacgtgctggatg
acaccagctggcacatggcggcgattgcgtccctggccgccaacgtcttgatgttcacgctgatctcgctgttcaccaatgccagccccgaagaaac
cagcgccgccgaagcctgcgcggtggacaacgtgcgccgcccgcaacgccgctcaatgctgtcggtgcagatcagcgatctgctgagcctggctgcg
cgctttgtgggtgaagaacgggcccagcaaagctttatccgtttcGCCTATCGCCAGGGTAAAGGCTTCAACCCCAACCAGAATGCCGACAACGATT
GGATCGCCCACACGGAACGCTTGCTGGCCGGTGTACTCGGTGCATCCTCGACCCGCGCAGTGGTAAAAGCAGCCATCGAAGGGCGGGAAATGCAGTT
GGAGGACGTCGTACGCATCGCCGACGAAGCGTCGGAAGTGCTGCAGTTCAACCGCGCGTTACTGCAAGGCGCCATCGAAAACATCACCCAAGGCATC
AGCGTGGTCGACCAGTCCCTCAAGCTGGTGGCCTGGAACCGGCGCTACCTGGAGCTGTTCAATTACCCCGACGGGCTGATCAGCGTGGGCCGGCCAA
TTGCCGACATCATCCGCTACAACGCCGAACGCGGGCTGTGCGGGCCGGGCGAGGCCGAAGTGCACGTGGCGCGACGCCTGCATTGGATGCGCCAGGG
CCGCGCGCACACGTCCGAGCGTTTATTCCCCAATGGCCGCGTGATCGAACTGATCGGCAACCCAATGCCCGGCGGCGGTTTCGTCATGAGCTTCACT
GACATTACCGCGTTCCGCGAAGCCGAACAGGCACTCACCGAAGCCAACGAAGGCCTGGAGCAACGGGTGACCGAGCGCACCCACGAGCTGTCCCAGC
TCAACGTGGCGCTCACTGACGCCAAGGGCGTGGCCGAATCTGCCAGCCAGTCGAAGACGCGCTTCCTGGCGGCCGTCAGCCATGACCTGATGCAGCC
GTTGAATGCTGCGCGCCTGTTCTCCGCCGCCCTCTCCCACCAGAACGACGGCCTGTCCAGCGAAGCCCGGCAATTGGTGCAACACCTGGACAGTTCG
CTGCGCTCTGCCGAAGACCTGATCAGCGACCTACTGGATATTTCTCGCCTGGAAAACGGCAAGATCAACCCACAGCGCCAGCCCTTTGTGCTCAATG
AGCTGTTCGACACCCTCGGCGCGGAATTCAAGGCGCTCGCCCAGGAGCAAGGTCTGCGCTTCCGCCTGCGCGGCAGCCGTTTGCGCGTCGACAGCGA
CATAAAGTTGTTACGACGGATTCTGCAGAATTTTCTCACCAACGCCTTCCGTTATGCCGACGGCCCGGTGCTGCTGGGCGTACGCCGACGTAAAGGC
GAGCTGTGCTTGGAAGTGTGGGACCGTGGCCCGGGTATCCCGCAGGATAAACAGAAGGTGATCTTCGAAGAATTCAAGCGCCTGGACAGCCACCAGA
CCCGCGCGGAGAAAGGCCTGGGACTTGGCTTGGCGATCGCCGACGGCCTGTGCCGCGTGCTGGACCACCGCTTGAGCGTGCGCTCATGGCCAGGCAA
GGGCAGCGTGTTCAGTGTGCGCGTGCCGCTGGCACGCAACCAGGCCACCCCGCTGGTCAAGACGCCGCAGGAAACCGGGCTGCCGTTGAGCGGTGCG
CAGGTGCTGTGCGTGGATAACGAAGAGAGCATCCTGATTGGCATGCGCAGCCTGTTGACGCGCTGGGGTTGCGAAGTGTGGACCGCCACCGACCAGG
CGCAATGCGCGGCGCTGCTGGCCGAGGGCGTGCGCCCGCAGCTGGCACTGGTGGATTACCACCTGGATCATGGCGAGACCGGTACTGAGTTGATGGG
CTGGCTGCGCGCGCAATTGGCGGAGCCGATTCCCGGCGTGGTCATCAGTGCCGACGGCCGCCCGGAAATGGTGGCCGAGGTGCATGCGGCGGGGCTG
GATTACCTGGCCAAGCCGGTGAAGCCGGCGGCGTTGCGAGCGTTGCTGAGTCGGCATCTGCCCCTTTAGGTCAGTGCCACATTTTGGTTTGTACCTA
TTCGGGCAGATGGGCCACGCCGTCGGAATCGGTCATCGCCCGCTCCAGCAGATCGGCCGGCAAGCTTTTACTCGCTCGCGCGCCGAGCAACCTCAAT
TGCTCGGTCCGGCTGAACCCAGCTTTCTTGTACAAAGTGGTCCCC

Aminoacid

MSFSLTQMLLISAAYLAALFGVAWISERGMIPRAIIRHPLTYTLSLGVYASAWAFYGTVGLAYQYGYGFLSSYLGVSGAFLLAPVLLYPILKITRTY
QLSSLADLFAFRFRSTWAGALTTIFMLIGVLPLLALQIQAVADSISILTREPVQHRVALAFCALITLFTIFFGSRHIATREKHEGLVFAIAFESVIK
LIAIGGVGLYALYGVFDGPQQLELWLLQNQTALAALHTPLQEGPWRTLLLVFFASAIVMPHMYHMTFTENLNPRSLVSASWGLPLFLLLMSLAVPLI
LWAGLKLGATTNPEYFTLGIGIAANSPALALLAYVGGLSAASGLIIVTTLALSGMALNHLVLPLYQPPAEGNIYRWLKWTRRALIVAIIMAGYGFYL
LLGAGQDLANLGIVAFVATLQFLPGVLSVLYWPTANRRGFIAGLLAGILVWIVTMLLPLVGNLQGFYIPLLNMIYVLDDTSWHMAAIASLAANVLMF

TLISLFTNASPEETSAAEACAVDNVRRPQRRSMLSVQISDLLSLAARFVGEERAQQSFIRFAYRQGKGFNPNQNADNDWIAHTERLLAGVLGASSTR
AVVKAAIEGREMQLEDVVRIADEASEVLQFNRALLQGAIENITQGISVVDQSLKLVAWNRRYLELFNYPDGLISVGRPIADIIRYNAERGLCGPGEA
EVHVARRLHWMRQGRAHTSERLFPNGRVIELIGNPMPGGGFVMSFTDITAFREAEQALTEANEGLEQRVTERTHELSQLNVALTDAKGVAESASQSK
TRFLAAVSHDLMQPLNAARLFSAALSHQNDGLSSEARQLVQHLDSSLRSAEDLISDLLDISRLENGKINPQRQPFVLNELFDTLGAEFKALAQEQGL
RFRLRGSRLRVDSDIKLLRRILQNFLTNAFRYADGPVLLGVRRRKGELCLEVWDRGPGIPQDKQKVIFEEFKRLDSHQTRAEKGLGLGLAIADGLCR
VLDHRLSVRSWPGKGSVFSVRVPLARNQATPLVKTPQETGLPLSGAQVLCVDNEESILIGMRSLLTRWGCEVWTATDQAQCAALLAEGVRPQLALVD
YHLDHGETGTELMGWLRAQLAEPIPGVVISADGRPEMVAEVHAAGLDYLAKPVKPAALRALLSRHLPL
