# Supplementary material for: Characterization of the CrbS/R Two-Component System in Pseudomonas fluorescens Reveals a New Set of Genes under Its Control and a DNA Motif Required for CrbR-Mediated Transcriptional Activation
Source: Front Microbiol. 2017 Nov 20;8:2287. doi: 10.3389/fmicb.2017.02287 (PMC5715377; doi:10.3389/fmicb.2017.02287)
Supplement: Supplementary file 2 [file Image2.PDF]

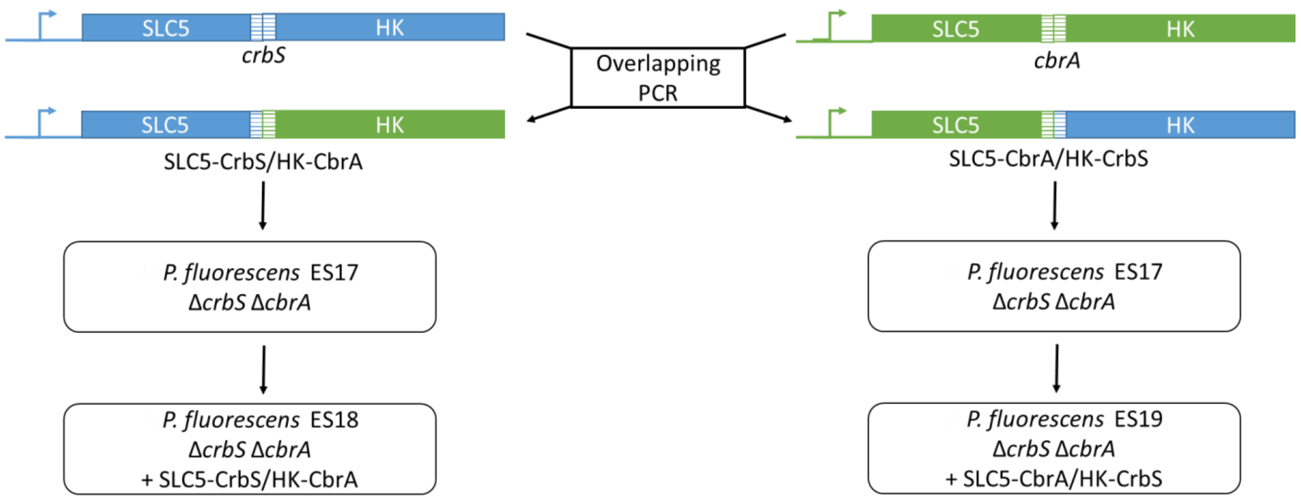

Figure S1. Schematic of the construction of *P.fluorescens* strains ES18 and ES19. Arrows represent the promoter region of each gene. The STAC domain is located in the region with the horizontal stripes pattern.The arginine used as a fusion point is represented by the line at the middle of the STAC domain. For the sake of clarity, line and box models are not scaled.

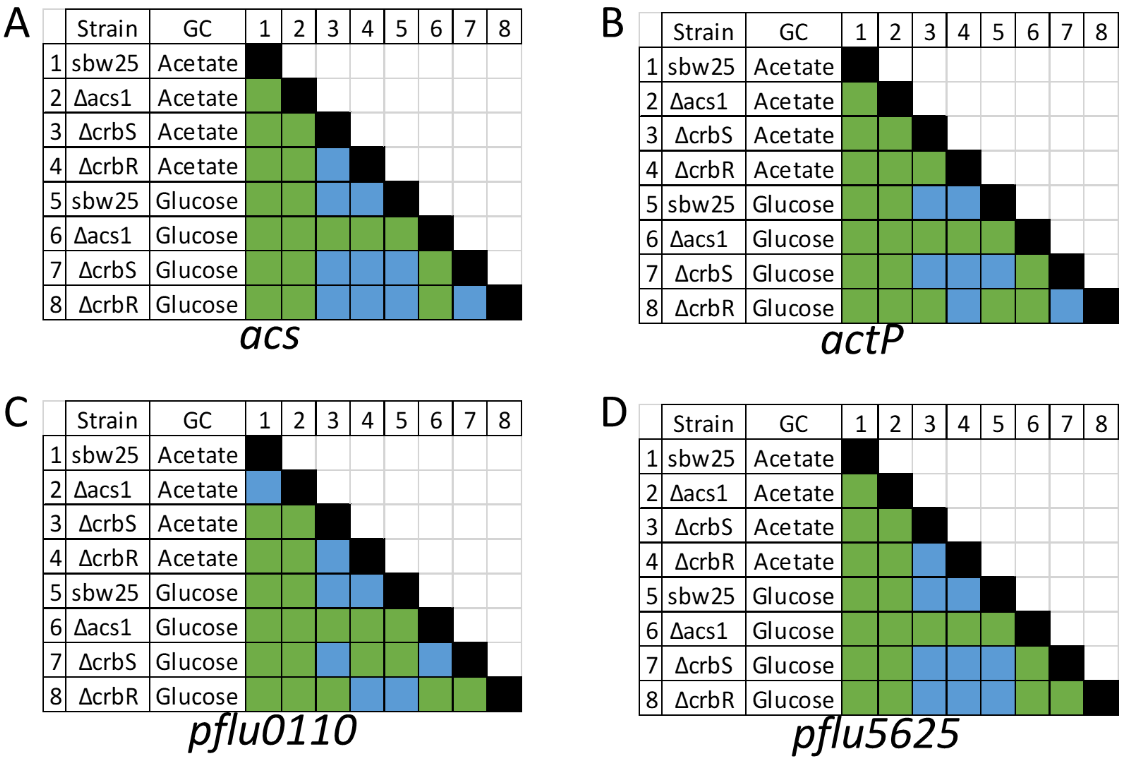

FigureS2. Comparison of means for uidA-transcriptional fusions of the A) *acs*, B)*actP*, C)*pflu0110* and D)*pflu5625* promoters depicted in figure 4. A one-way ANOVA with a Tukey post-test was used to determine statistical significance. Green Box - No significant difference between means P< 0.005; Blue Box – No significant difference between means; GC – Growth conditions.
